# CYP3A4, CYP3A5, and CYP4F2 Polymorphisms and Bleeding Risk in Ticagrelor-Based Dual Antiplatelet Therapy

**DOI:** 10.3390/medicina62061202

**Published:** 2026-06-22

**Authors:** Sonja Dakić, Zoran Perišić, Svetlana Apostolović, Tomislav Kostić, Goran Koraćević, Tatjana Jevtović, Boris Đinđić, Nikola Stefanović, Danijela Đorđević-Radojković, Bojan Maričić, Dragana Stanojević, Maša Jović, Jelena Perišić, Tamara Filipović

**Affiliations:** 1 Clinic for Cardiology, University Clinical Center of Nis, 18000 Nis, Serbia; perisiczoran@icloud.com (Z.P.); drapostolovic@gmail.com (S.A.); tomislav.kostic1977@gmail.com (T.K.); gkoracevic@yahoo.com (G.K.); boris_dj@yahoo.com (B.Đ.); neladjr@gmail.com (D.Đ.-R.); bokimaricic@gmail.com (B.M.); draganadrstanojevic@gmail.com (D.S.); jelenaperisic@icloud.com (J.P.); tamaricak97@gmail.com (T.F.); 2 Faculty of Medicine, University of Nis, Bulevar Dr Zorana Đinđića 81, 18000 Nis, Serbia; jovic.98.masa@gmail.com; 3 Department of Biochemistry, Faculty of Medicine, University of Nis, 18000 Nis, Serbia; tjevtovic@yahoo.com; 4 Department of Pathophysiology, Faculty of Medicine, University of Nis, 18000 Nis, Serbia; 5 Department of Pharmacy, Faculty of Medicine, University of Nis, 18000 Nis, Serbia; nikola.z.stefanovic@gmail.com

**Keywords:** ticagrelor, acute coronary syndrome, bleeding, pharmacogenetics, CYP3A4, CYP3A5, CYP4F2, dual antiplatelet therapy, BARC, Firth regression

## Abstract

*Background and Objectives*: Ticagrelor reduces ischemic events in acute coronary syndrome (ACS) but increases bleeding risk. Clinical predictors of bleeding are well established; the contribution of cytochrome P450 polymorphisms involved in ticagrelor metabolism remains uncertain, with conflicting reports in the literature. We examined the association of CYP3A4* 22 (rs 35599367), CYP3A5* 3 (rs 776746), and CYP4F2 (rs3093135) with bleeding in a Serbian ACS cohort. *Materials and Methods*: This prospective, single- center observational study enrolled 105 consecutive ACS patients undergoing percutaneous coronary intervention (PCI) or medical management after coronary angiography and receiving dual antiplatelet therapy (DAPT) with acetylsalicylic acid and ticagrelor at the University Clinical Center Niš between January 2024 and the end of May 2025. Bleeding events occurring during the index hospitalization and the six-month follow-up were classified according to the Bleeding Academic Research Consortium (BARC) criteria. Genotyping used TaqMan assays. Associations with bleeding were assessed using Firth’s penalized logistic regression, with multivariable adjustment for age and renal function. Severity-stratified analyses and gradient-boosted machine learning (XGBoost with SHAP) were performed as exploratory analyses. *Results*: Thirteen patients (12.4%) experienced bleeding (nine minor [BARC 1/2], four major [BARC 3/5]). Age ≥ 75 years (univariable OR 7.62, *p* = 0.001) and eGFR < 60 mL/min/1. 73 m ^2^ (OR 3.68, *p* = 0.006) were the strongest predictors. CYP3A5 *1 carrier status was univariably associated with bleeding (OR 4.16, *p* = 0.043) but did not remain significant after adjustment for age and renal function, and *1 carriers were significantly older and more likely to have impaired renal function. No genotype was associated with major (BARC 3/5) bleeding. The apparent effect was concentrated in minor bleeding (BARC 1/2 rate: 30.8% versus 5.5%), with no major events among *1 carriers. CYP 3 A 4* 22 (OR 1.37, *p* = 0.109) and CYP 4 F 2 (OR 1.17, *p* = 0.111) showed no association. Machine-learning analyses confirmed eGFR and age as the dominant predictors. *Conclusions*: In this Serbian ACS cohort, clinical factors—particularly advanced age and impaired renal function—dominated the prediction of bleeding risk. The CYP3A5 signal was largely explained by baseline imbalances in age and renal function. CYP 3 A 4* 22 and CYP 4 F 2 polymorphisms did not contribute additional predictive information. Preemptive genotyping for these variants is unlikely to materially improve bleeding-risk assessment beyond standard clinical evaluation in patients of this type.

## 1. Introduction

Acute coronary syndrome (ACS) remains among the most common reasons for hospital admission and a leading cause of cardiovascular mortality worldwide [1]. Dual antiplatelet therapy (DAPT) combining acetylsalicylic acid with a P2Y12 receptor antagonist is the standard pharmacological strategy for patients undergoing percutaneous coronary intervention (PCI); current European Society of Cardiology guidelines list ticagrelor and prasugrel as preferred agents over clopidogrel for most patients with ACS [2]. Ticagrelor differs mechanistically from the older thienopyridines in several ways: it binds the P2Y12 receptor reversibly rather than irreversibly, it does not require metabolic activation, and its onset of platelet inhibition is faster and more consistent across patients [3]. In the pivotal PLATO trial of 18,624 ACS patients, ticagrelor reduced the composite of cardiovascular death, myocardial infarction, or stroke from 11.7% to 9.8% over twelve months compared with clopidogrel [4]. The same trial documented an unavoidable trade-off: rates of non-CABG major bleeding were higher with ticagrelor (4.5% versus 3.8%), and the cumulative bleeding burden during prolonged DAPT contributes substantially to long-term treatment risk, particularly in elderly and frail patients.

The risk of bleeding during antiplatelet therapy is not evenly distributed among patients. Several well-validated clinical scores—most prominently PRECISE-DAPT [5] and the Academic Research Consortium’s high-bleeding-risk consensus criteria [6]—converge on a similar shortlist of predictors. Advanced age, impaired renal function (typically defined as an eGFR < 60 mL/min/1.73 m^2^ or documented chronic kidney disease), anemia, prior major bleeding, and concomitant anticoagulation account for most of the inter-individual variability observed in clinical practice. PRECISE-DAPT incorporates age, creatinine clearance, hemoglobin, white blood cell count, and prior spontaneous bleeding into a five-item score with an area under the receiver operating characteristic curve of approximately 0.70 to 0.73 for one-year major bleeding in external validation cohorts [5]. The performance of these scores has been broadly consistent across European, Asian, and Latin American populations, suggesting that the underlying clinical biology of bleeding on DAPT is reasonably universal. What remains less certain is whether pharmacogenetic variation—that is, inherited differences in how individuals process the antiplatelet drug itself—adds clinically meaningful information beyond these clinical variables.

The case for a pharmacogenetic contribution to ticagrelor’s bleeding risk rests on its hepatic metabolism. Ticagrelor is converted in the liver to the active metabolite AR-C124910XX, which has platelet-inhibitory potency comparable to the parent drug and accounts for roughly thirty to forty percent of total systemic exposure [3,7]. This conversion is catalyzed almost entirely by cytochrome P450 3A enzymes, with CYP3A4 as the dominant catalytic isoform and CYP3A5 contributing a quantitatively smaller—but not negligible—share [8]. Both enzymes are highly polymorphic. Loss-of-function or reduced-function variants would, in principle, slow ticagrelor clearance, raise circulating drug levels, and intensify the platelet-inhibitory effect—and, by the same chain of reasoning, shift the bleeding–thrombosis balance toward more bleeding. The relevant question is whether this pharmacokinetic logic translates into a clinically detectable outcome signal at the magnitude of genetic effect observed in real patients.

The strongest a priori candidate for a clinically meaningful pharmacogenetic effect on ticagrelor metabolism is CYP3A4*22 (rs35599367, c.522-191C>T). The variant is an intronic C>T substitution in intron 6 of CYP3A4 that reduces hepatic CYP3A4 mRNA expression by roughly half and decreases the catalytic activity of the encoded enzyme [9]. In a controlled pharmacokinetic study of healthy Finnish volunteers, Holmberg and colleagues found that CYP3A4*22 carriers had approximately 60% higher ticagrelor exposure (area under the concentration–time curve) and substantially greater platelet inhibition 24 h after dosing than wild-type controls [10]. Whether this pharmacokinetic effect translates into measurable clinical bleeding has been examined in two outcome studies with discordant results. A Finnish register-based cohort of 368 patients with ischemic heart disease reported a hazard ratio of 3.74 (95% CI 1.26–11.05) for bleeding among *22 carriers [11], but a sub-study of the POPular Genetics randomized trial—by far the largest ticagrelor pharmacogenetic dataset to date, with 1281 STEMI patients—found no association of *22 with either the bleeding (adjusted HR 0.93) or the thrombotic endpoint [12]. The picture is therefore inconclusive, and the relative weight to attach to a small-cohort positive register study versus a substantially larger trial sub-study with a null finding remains a matter of judgment.

CYP3A5 is the second metabolic player. The major loss-of-function variant, CYP3A5*3 (rs776746, c.6986A>G), introduces an intronic splice defect that essentially abolishes functional enzyme expression in homozygous carriers [13]; only individuals carrying at least one wild-type *1 allele (“CYP3A5 expressors”) produce appreciable amounts of CYP3A5 protein in the liver. In European populations, approximately 80–90% of individuals are CYP3A5 *3/*3 non-expressors, whereas in African populations the wild-type *1 allele is more common. Despite the well-characterized effect of CYP3A5*3 on the disposition of several substrates—most notably tacrolimus and certain calcineurin inhibitors—the available evidence for a clinically relevant effect on ticagrelor metabolism is weak. Pharmacokinetic studies in healthy Chinese subjects [14] and the controlled crossover study by Holmberg and colleagues in Finland [10] both failed to detect a significant effect of CYP3A5*3 on ticagrelor exposure or on-treatment platelet inhibition. The POPular Genetics sub-study likewise reported no clinical effect of CYP3A5 expressor status on bleeding or thrombotic events [12]. The plausible biological reason is that CYP3A5’s contribution to total ticagrelor clearance is small enough that loss-of-function in the minor isoform is fully compensated by intact CYP3A4 activity; the metabolic system is, by design, redundant.

The role of the CYP4F2 rs3093135 A>T polymorphism is mechanistically more indirect. CYP4F2 is a fatty-acid omega-hydroxylase that catalyzes the conversion of arachidonic acid to 20-hydroxyeicosatetraenoic acid (20-HETE), a bioactive eicosanoid with vasoactive activity [15]. Early mechanistic models suggested that 20-HETE may inhibit platelet aggregation by antagonizing thromboxane receptor signaling; however, more recent evidence indicates that 20-HETE-mediated platelet effects are complex and may involve downstream lipid metabolites rather than direct receptor antagonism [16,17]. A Lithuanian cohort study reported that T-allele carriers of rs3093135 had lower platelet aggregation values, suggesting reduced platelet reactivity [18]; a subsequent paper from the same investigators, examining a partially overlapping cohort, instead reported an association with increased bleeding [19]. A larger Finnish register study found no signal [11]. The relevance of CYP4F2 rs3093135 to ticagrelor-related bleeding in any specific patient population remains, at best, an open empirical question.

Most published pharmacogenetic literature on ticagrelor derives from pharmacokinetic studies of small numbers of healthy volunteers or from outcome studies conducted in Western European, Scandinavian, or East Asian populations. South-eastern European cohorts, including Serbian and other Balkan populations, are systematically underrepresented in this literature, even though the genetic ancestry of these populations is heterogeneous and not necessarily well approximated by Northern or Western European reference samples. Whether the modest and inconsistent pharmacogenetic signals observed elsewhere replicate in a Serbian ACS cohort remains unknown given the available data. The clinical case for studying this question lies in the practical management of antiplatelet therapy: any pretreatment genotype that meaningfully improves bleeding-risk prediction beyond what age and renal function already provide would be useful, but the magnitude of any such incremental gain—and the populations in which it would apply—needs to be empirically established rather than assumed.

Against this background, the present study had two pre-specified aims. First, to characterize clinical and pharmacogenetic predictors of bleeding in a consecutive cohort of 105 Serbian ACS patients receiving ticagrelor-based DAPT after PCI or medical management of ACS, using validated clinical risk variables alongside three pharmacogenetically relevant polymorphisms (CYP3A4*22 rs35599367, CYP3A5*3 rs776746, and CYP4F2 rs3093135). Second, to quantify the independent contribution of each polymorphism to bleeding risk after adjustment for the dominant clinical predictors, using statistical methods appropriate for the sparse-event setting (Firth’s penalized logistic regression) [20]. As exploratory analyses, we also examined whether the effect of any candidate polymorphism differed by BARC severity and whether a non-linear machine-learning approach altered the inferential picture obtained from regression.

## 2. Materials and Methods

### 2.1. Study Design and Patient Population

This prospective, single-center observational cohort study was conducted at the Clinic for Cardiology of the University Clinical Centre Niš, Serbia, from January 2024 through the end of May 2025. Consecutive adult patients admitted with ACS–ST-segment elevation myocardial infarction (STEMI), non-ST-segment elevation myocardial infarction (NSTEMI), or unstable angina, who were initiated on DAPT with acetylsalicylic acid plus ticagrelor, were screened for eligibility. ACS was diagnosed by the attending cardiologist in accordance with current European Society of Cardiology guidelines.

Inclusion criteria were: age ≥ 18 years; angiographically confirmed ACS treated with PCI or continued medical management after coronary angiography; initiation of ticagrelor (180 mg loading dose followed by 90 mg twice daily) on top of acetylsalicylic acid (300 mg loading, 100 mg daily maintenance) during the index hospitalization; and written informed consent. Exclusion criteria were: prior switch from another P2Y12 inhibitor without a defined washout period; known hypersensitivity to ticagrelor; severe hepatic impairment (Child-Pugh class C); active malignancy; severe renal impairment (eGFR < 15 mL/min/1.73 m^2^ or maintenance dialysis); planned coronary artery bypass grafting (CABG) after coronary angiography; and unwillingness or inability to provide consent. A total of 105 patients met all eligibility criteria and were included in the final analysis.

The study was conducted in accordance with the Declaration of Helsinki (revised 2013) and approved by the Ethics Committee of the University Clinical Center Niš (approval number: 38349/15; approval date: 15 December 2023). All participants provided written informed consent prior to enrollment and to genetic testing, including explicit consent to the use of their genotype and clinical data for research publication in anonymized form. The genetic data have been pseudonymized, are stored on encrypted institutional servers, and are accessible only to the research team. The full anonymized dataset is available from the corresponding author upon reasonable request, subject to compliance with the Serbian Personal Data Protection Act and applicable institutional policies.

### 2.2. Clinical Data Collection and Follow-Up

Demographic, anthropometric, clinical, and procedural data were extracted by a single investigator from patients’ medical records (including hospital discharge summaries) and entered into a standardized case report form. Recorded variables included age, sex, body mass index, smoking status, ACS subtype at presentation (STEMI, NSTEMI, or unstable angina), arterial hypertension, type 2 diabetes mellitus, prior coronary artery disease, prior chronic kidney disease, prior bleeding events, and prior thrombotic events. Procedural variables included arterial access route (femoral or radial), number of treated coronary vessels, and number of stents implanted. Coronary procedures were performed predominantly via femoral access using 6-French sheaths; haemostasis was achieved by manual compression or a vascular closure device at the operator’s discretion. All patients undergoing PCI received periprocedural unfractionated heparin according to the institutional protocol, and glycoprotein IIb/IIIa receptor inhibitors were administered only as bail-out therapy at the operator’s discretion rather than routinely. Concomitant medications at discharge were recorded, including a β-blocker, an angiotensin-converting enzyme inhibitor, a mineralocorticoid receptor antagonist, a sodium–glucose co-transporter 2 inhibitor, a calcium-channel blocker, a statin, and a proton-pump inhibitor.

Laboratory parameters obtained on admission included complete blood count (hemoglobin, hematocrit, white blood cell count, platelet count), serum creatinine, serum urea, aspartate aminotransferase, alanine aminotransferase, C-reactive protein, fasting glucose, total cholesterol, LDL cholesterol, HDL cholesterol, and triglycerides. Estimated glomerular filtration rate (eGFR) was calculated using the Modification of Diet in Renal Disease (MDRD) equation [21]. Left ventricular ejection fraction was measured by transthoracic echocardiography during the index hospitalization.

Patients were followed for six months from the index admission, with scheduled clinical visits at one and six months, and additional unscheduled contact whenever a clinical event was reported. Patients who did not attend scheduled visits were contacted by telephone. Bleeding events occurring during the index hospitalization were captured prospectively from the inpatient record; events occurring after discharge were captured at scheduled visits and, when available, confirmed against external clinical documentation.

### 2.3. Outcome Definitions

The primary outcome was any bleeding event during the observation period (index hospitalization plus six-month follow-up). Bleeding events were classified using the standardized BARC criteria [22]. For severity-stratified analysis, BARC types 1 and 2 were grouped as “minor” bleeding (clinically apparent bleeding that does not require intervention or hospitalization), and BARC types 3a, 3b, 3c, and 5 were grouped as “major” bleeding (events associated with a hemoglobin decrease of ≥30 g/L, requiring medical intervention or transfusion, or causing death). The anatomical location of each bleeding event was recorded as access-site, gastrointestinal, genitourinary, intracranial, or other. Bleeding severity was adjudicated by a single clinical investigator with access to the complete medical record.

### 2.4. DNA Extraction and Genotyping

Fasting whole-blood samples were collected from each patient at enrollment in EDTA-containing tubes and stored at −20 °C until processing. Genomic DNA was extracted from 200 μL of whole blood using the PureLink Genomic DNA Mini Kit (Invitrogen, Thermo Fisher Scientific, Carlsbad, CA, USA) following the manufacturer’s instructions. Extracted DNA was diluted to a working concentration of 5 ng/µL for real-time PCR, and the integrity of each sample was confirmed by ultraviolet spectrophotometry.

Three single-nucleotide polymorphisms were genotyped: CYP3A5*3 (rs776746), CYP3A4*22 (rs35599367), and CYP4F2 rs3093135 (A>T). Genotyping was performed using TaqMan Drug Metabolism Genotyping Assays (Applied Biosystems, Carlsbad, CA, USA) with assay identifiers C__26201809_30 for CYP3A5*3, C__59013445_10 for CYP3A4*22, and C__27482167_10 for CYP4F2 rs3093135. Reactions were run on a 7500 Fast Real-Time PCR System (Applied Biosystems, Foster City, CA, USA) according to the manufacturer’s instructions. The FAM fluorescent dye labeled the variant (polymorphic) allele, and the VIC fluorescent dye labeled the wild-type allele for each assay. Allelic discrimination was performed using the system software and verified by visual inspection of the allelic-discrimination plots: homozygous genotypes formed distinct clusters along a single fluorescence axis, while heterozygous samples clustered between the VIC and FAM signals. Samples with ambiguous fluorescence patterns were re-extracted and re-analyzed; samples that failed to genotype on the second attempt would have been excluded, although no such exclusions were required in the final cohort.

An additional polymorphism, CYP3A4*1G (rs2242480), was originally genotyped in this cohort but was excluded from the present analysis. The variant was previously designated a CYP3A4 star allele based on the intronic c.1026+12G>A substitution; however, the Pharmacogene Variation (PharmVar) Consortium has since reconsidered this designation because the same variant occurs on multiple haplotype backgrounds and no consistent functional effect on CYP3A4 activity has been demonstrated across studies. Consistent with current PharmVar guidance [23], we therefore restricted the present analysis to the three polymorphisms with established or robust functional annotation. CYP3A4*1G genotypes will be reported separately as part of a doctoral thesis project.

### 2.5. Statistical Analysis

Continuous variables are presented as medians with interquartile ranges (IQRs), and categorical variables as counts and percentages. Baseline comparisons between patients with and without bleeding events used the Mann–Whitney U test for continuous variables and Fisher’s exact test for binary categorical variables; the Pearson chi-square test was used for multilevel categorical variables when the smallest expected cell count was at least 5. Two-tailed *p* values are reported throughout, and a significance threshold of 0.05 was applied to all hypothesis tests. No correction for multiple testing was applied, consistent with the exploratory nature of the pharmacogenetic analyses; readers are advised to weight nominal *p* values accordingly.

Hardy–Weinberg equilibrium for each polymorphism was tested using Pearson’s chi-square test and verified with the exact procedure of Wigginton, Cutler, and Abecasis [24]. Minor allele frequencies were calculated from observed genotype counts and compared with published European reference data. All three polymorphisms were modeled under a dominant inheritance pattern, comparing carriers of at least one minor allele with homozygotes for the major allele. The dominant model was selected a priori for two reasons: first, the rarity of homozygous variant genotypes in our cohort (no CYP3A5 *1/*1 and no CYP3A4*22 T/T) precluded meaningful additive or recessive modeling for these two polymorphisms; second, both CYP3A5*3 and CYP3A4*22 have functional evidence supporting dominant-effect modeling in clinical pharmacogenetic studies [9,13].

Given the rare-event setting (13 bleeding events among 105 patients), the association analyses used Firth’s penalized maximum-likelihood approach throughout, rather than ordinary maximum-likelihood logistic regression [25,26]. Firth’s penalty, derived from Jeffreys’s invariant prior, removes the first-order asymptotic bias of the maximum-likelihood estimator and yields finite, interpretable parameter estimates and confidence intervals even when complete or quasi-complete separation occurs—a situation that arose, for example, in the BARC-major-bleeding stratum, where no events were observed among CYP3A5 *1 carriers. Inference on individual coefficients used the penalized likelihood-ratio test rather than the Wald test, which can be unreliable in sparse-data settings [27]. All confidence intervals reported are profile penalized-likelihood intervals derived from the same penalized likelihood as the likelihood-ratio tests, so that interval coverage and hypothesis tests are mutually consistent: a 95% interval excludes the null value of 1.0 if and only if the corresponding penalized likelihood-ratio test *p* value is below 0.05. The Firth procedure was implemented in Python (version 3.12) using the iterative reweighted least squares formulation described by Heinze and Schemper; convergence was assessed by monitoring changes in the coefficient vector of less than 10^−7^ across iterations.

Univariable Firth’s penalized logistic regression was performed for each candidate predictor against the primary composite bleeding outcome. Two pre-specified multivariable models were then fitted to estimate the independent effect of CYP3A5 *1 carrier status after adjustment for the dominant clinical predictors of bleeding. Model A used continuous predictors (age, eGFR), prior chronic kidney disease, and CYP3A5*1 carrier status. Model B used clinically interpretable binary cut-points (age ≥ 75 years; eGFR < 60 mL/min/1.73 m^2^) and CYP3A5 *1 carrier status. The number of covariates was deliberately kept small to maintain an events-per-predictor ratio of at least 3, recognizing that this is below the conventional ratio of 10 commonly recommended for unpenalized logistic regression [27]; Firth penalization mitigates but does not eliminate the resulting instability. CYP3A4*22 and CYP4F2 polymorphisms were not retained in the multivariable models because neither showed a univariable association with bleeding in our cohort.

Baseline characteristics across CYP3A5 genotype groups were compared in a pre-specified analysis to assess potential confounding of the genotype–outcome association. A pre-specified sensitivity analysis was conducted by refitting the principal CYP3A5 association after excluding patients with admission C-reactive protein levels above 50 mg/L (*n* = 6) to assess robustness to the influence of marked systemic inflammation, which may secondarily perturb both ticagrelor pharmacokinetics and bleeding susceptibility.

Severity-stratified analyses were conducted by repeating the univariable and parsimonious multivariable Firth models, with BARC 1/2 (minor) and BARC 3/5 (major) bleeding as separate binary outcomes. The events-per-predictor ratios in these stratified analyses (approximately 3 for minor and 1.3 for major bleeding) are substantially below conventional thresholds, so these results are reported as exploratory.

To complement the regression analysis with a non-linear, non-parametric perspective, gradient-boosted tree classifiers (XGBoost, version 2.0) were trained with stratified 5-fold cross-validation, repeated 20 times, yielding 100 out-of-fold area under the receiver operating characteristic (AUC) estimates per model configuration. Hyperparameters were fixed in advance (maximum depth = 3, learning rate = 0.05, subsample = 0.8, colsample_bytree = 0.8, 200 boosting rounds with early stopping based on cross-validated log-loss) to limit overfitting in this small-sample setting. Five model configurations were compared: a parsimonious clinical model (age, eGFR, prior chronic kidney disease); the parsimonious model with CYP3A5 *1 carrier status added; a broader clinical model (eight features); the broader model with CYP3A5 added; and a parsimonious clinical model with all three genotypes added. Feature importance was estimated using SHAP (SHapley Additive exPlanations) values [28]; cross-validated AUC distributions were compared between paired model configurations using the paired Wilcoxon signed-rank test. The machine-learning analysis was implemented in Python 3.12 using XGBoost, scikit-learn 1.4, and shap 0.45.

## 3. Results

### 3.1. Baseline Characteristics of the Study Cohort

A total of 105 consecutive ACS patients who received ticagrelor as part of DAPT were included in the analysis. The median age was 64.0 years (IQR 54.0–70.0), and 79 patients (75.2%) were male. The most common clinical presentation was STEMI (74 patients, 70.5%), followed by NSTEMI (22 patients, 21.0%) and unstable angina (nine patients, 8.6%). Arterial hypertension was the most prevalent comorbidity (84.8%), followed by type 2 diabetes mellitus (38.1%), prior coronary artery disease (28.6%), and chronic kidney disease (10.5%). Of the 105 patients, 98 (93.3%) underwent PCI with stent implantation, and femoral arterial access was used in 101 patients (96.2%). All patients received a statin and a proton-pump inhibitor during hospitalization. The complete baseline clinical, laboratory, and procedural characteristics—overall and stratified by the occurrence of any bleeding event—are presented in Table 1.

### 3.2. Bleeding Outcomes

During the observation period (index hospitalization and 6-month follow-up), a bleeding event of any severity was recorded in 13 patients (12.4%). Eleven events (10.5%) occurred during the index hospitalization, and two (1.9%) during the 6-month follow-up; no patient experienced bleeding at both time points. According to the BARC criteria, four events (3.8%) were major (BARC type 3 or 5) and nine (8.6%) were minor (BARC type 1 or 2). The most frequent bleeding sites were the arterial puncture site (five events), the genitourinary tract (hematuria, four events), the gastrointestinal tract (one event with melena), and other locations (two events).

### 3.3. Genotype Distribution and Hardy–Weinberg Equilibrium

Observed genotype counts, minor allele frequencies (MAF), and Hardy–Weinberg equilibrium (HWE) test results for the three polymorphisms are summarized in Table 2. All three polymorphisms were in HWE, as confirmed by both the Pearson chi-square test and the exact test of Wigginton et al. (all *p* > 0.05). The observed MAFs were consistent with those previously reported for European populations: 3.81% for CYP3A4*22 (T), comparable to the Finnish estimate of approximately 3.0% [11] and the Central European value of around 5% [29]; 6.19% for CYP3A5*1; and 24.29% for CYP4F2 rs3093135 (T). No CYP3A4*22 T/T homozygotes or CYP3A5*1/*1 homozygotes were observed in the cohort, consistent with the expected genotype counts under HWE given the low MAFs.

**Figure 1:**
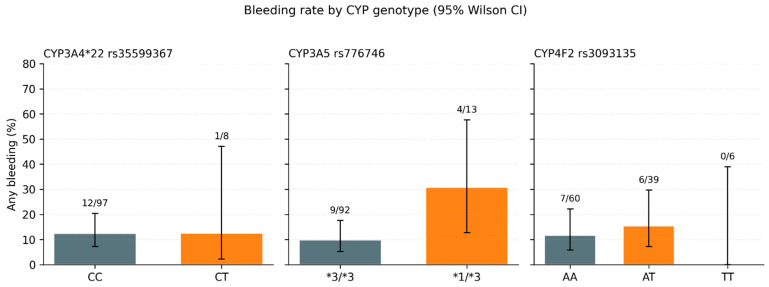
Unadjusted proportion of patients who experienced any bleeding event (in-hospital or during the 6-month follow-up), stratified by genotype for each of the three CYP polymorphisms investigated. Error bars represent 95% Wilson confidence intervals for each proportion. Observed event counts (number of events/total number of patients in the genotype group) are annotated above each bar.

Unadjusted bleeding rates by genotype are shown in Figure 1. A higher bleeding rate was observed among CYP3A5 *1/*3 heterozygotes (4/13, 30.8%) than among *3/*3 homozygotes (9/92, 9.8%), whereas no clear genotype-dependent gradient was apparent for the CYP3A4*22 or CYP4F2 rs3093135 polymorphisms.

### 3.4. Univariable Associations with Any Bleeding

**Figure 2:**
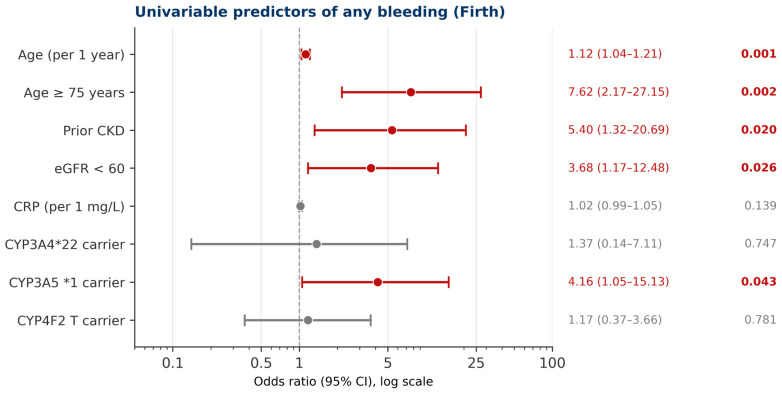
Forest plot of univariable odds ratios with 95% confidence intervals for the principal clinical and genetic predictors of any bleeding, estimated by Firth’s penalized logistic regression. Effect estimates are plotted on a logarithmic scale; the dashed vertical line denotes an odds ratio of 1.0 (no association). Red markers and bold text indicate associations that are statistically significant at the *p* < 0.05 level by the penalized likelihood-ratio test; gray markers indicate non-significant associations.

Univariable associations between each candidate predictor and any bleeding were assessed using Firth’s penalized logistic regression, which yields unbiased effect estimates and finite confidence intervals in sparse-event settings. The principal results are summarized in Table 3 and shown as a forest plot in Figure 2; the complete univariable analysis is presented in Table S1 of the Supplementary Materials.

Clinical predictors dominated the univariable signal. Age—analyzed either as a continuous variable (OR 1.12 per year, 95% CI 1.04–1.21; *p* = 0.001) or as the binary cut-point of ≥75 years (OR 7.62, 95% CI 2.17–27.15; *p* = 0.002)—and indicators of impaired renal function—prior chronic kidney disease (OR 5.40, 95% CI 1.32–20.69; *p* = 0.020), eGFR as a continuous variable (OR 0.96 per 1 mL/min/1.73 m^2^, 95% CI 0.93–0.99; *p* = 0.012), and eGFR < 60 mL/min/1.73 m^2^ (OR 3.68, 95% CI 1.17–12.48; *p* = 0.026)—emerged as the strongest predictors of bleeding.

Among the three polymorphisms investigated, only CYP3A5 *1 carrier status (*1/*3 versus *3/*3) showed a statistically significant univariable association with any bleeding (OR 4.16, 95% CI 1.05–15.13; *p* = 0.043). Neither CYP3A4*22 carrier status (OR 1.37, 95% CI 0.14–7.11; *p* = 0.747) nor CYP4F2 rs3093135 T-allele carrier status (OR 1.17, 95% CI 0.37–3.66; *p* = 0.781) was significantly associated with the primary outcome. As discussed below, the apparent association with CYP3A5 must be interpreted in the context of substantial baseline imbalances in age and renal function across CYP3A5 genotype groups.

### 3.5. Multivariable Models of Bleeding Risk

Before constructing multivariable models, baseline clinical characteristics were compared between CYP3A5 *3/*3 homozygotes and CYP3A5 *1 carriers (Table S2). This comparison revealed a significantly higher prevalence of prior chronic kidney disease (30.8% versus 7.6%; *p* = 0.029) and of eGFR < 60 mL/min/1.73 m^2^ (61.5% versus 29.3%; *p* = 0.029) among CYP3A5 *1 carriers. CYP3A5 *1 carriers also had a numerically lower median eGFR (49.7 versus 72.5 mL/min/1.73 m^2^; *p* = 0.079) and a numerically higher proportion of patients aged ≥75 years (30.8% versus 12.0%; *p* = 0.089). These imbalances suggest that the unadjusted association between CYP3A5 *1 carrier status and bleeding could be partly attributable to co-occurring clinical risk factors. To address this, two multivariable Firth’s penalized logistic regression models were constructed.

**Figure 3:**
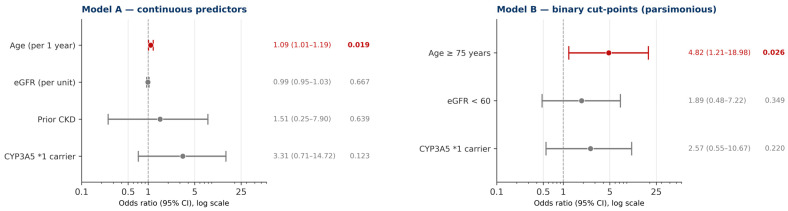
Forest plot of adjusted odds ratios from two multivariable Firth’s penalized logistic regression models. (**Left**) Model A with continuous age and eGFR, prior chronic kidney disease, and CYP3A5 *1 carrier status. (**Right**) Model B with binary cutpoints (age ≥ 75 years and eGFR < 60 mL/min/1.73 m^2^) and CYP3A5 *1 carrier status. Red markers indicate predictors that remain statistically significant after mutual adjustment (*p* < 0.05, as determined by a penalized likelihood-ratio test). The dashed vertical line denotes an adjusted odds ratio of 1.0 (no association).

Model A included age, eGFR, prior chronic kidney disease, and CYP3A5 *1 carrier status as continuous and binary predictors, respectively. Model B used clinically interpretable binary cut-points (age ≥ 75 years and eGFR < 60 mL/min/1.73 m^2^) and CYP3A5 *1 carrier status. Both models retained the CYP3A4*22 and CYP4F2 genotypes only in univariable analysis, as neither was associated with the outcome. Detailed results are presented in Table 4 and visualized in Figure 3.

Across both models, age (or age ≥ 75 years) remained an independent predictor of bleeding: adjusted OR 1.09 per year (95% CI 1.01–1.19; *p* = 0.019) in Model A and adjusted OR 4.82 (95% CI 1.21–18.98; *p* = 0.026) in Model B. CYP3A5 *1 carrier status was attenuated to non-significance after adjustment in both models (Model A: adjusted OR 3.31, 95% CI 0.71–14.72; *p* = 0.123; Model B: adjusted OR 2.57, 95% CI 0.55–10.67; *p* = 0.220); the wide confidence intervals, both crossing one, reflect the limited number of bleeding events (*n* = 13) and the substantial overlap between CYP3A5 *1 carrier status and the renal-function predictors. Indicators of renal function (eGFR or eGFR < 60 mL/min/1.73 m^2^) and prior chronic kidney disease lost statistical significance after adjustment for age, consistent with the well-known correlation between age and renal function in this clinical setting. These results indicate that advanced age is the dominant independent predictor of bleeding, that the renal-function indicators are partly collinear with age, and that the univariable CYP3A5 association does not survive adjustment—consistent with confounding by the older, more renally impaired profile of *1 carriers rather than an independent pharmacological effect.

### 3.6. Bleeding Outcomes Stratified by CYP3A5 Genotype and BARC Severity

**Figure 4:**
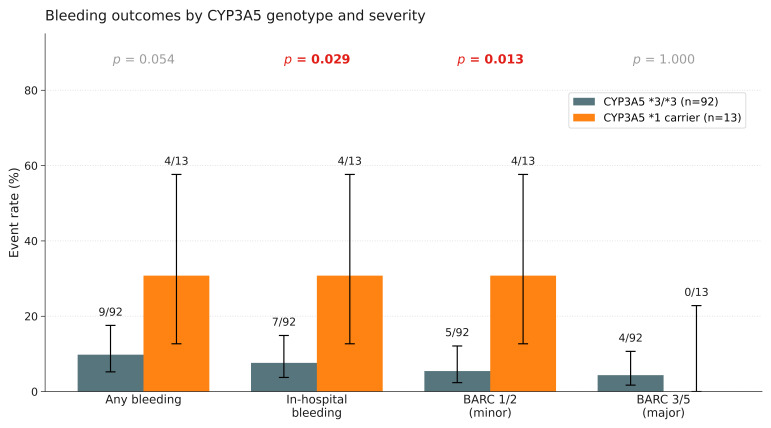
Unadjusted bleeding event rates by CYP3A5 genotype across four outcome categories: any bleeding (composite of in-hospital and 6-month events), in-hospital bleeding only, BARC type 1/2 (minor) bleeding, and BARC type 3/5 (major) bleeding. Error bars represent 95% Wilson confidence intervals. Event counts (events/total) are annotated above each bar; *p* values from Fisher’s exact test are shown above each pair of bars, with statistically significant differences (*p* < 0.05) in bold red.

Although CYP3A5 *1 carrier status was associated with the composite bleeding outcome, stratification by BARC severity revealed a clear dissociation between minor and major bleeding (Figure 4 and Table 5). CYP3A5 *1 carriers experienced minor bleeding (BARC type 1 or 2) in 30.8% of cases (4/13), compared with only 5.4% (5/92) of *3/*3 homozygotes (Fisher exact OR 7.73, 95% CI 1.75–34.08; *p* = 0.013). In contrast, all four major bleeding events (BARC type 3 or 5) occurred in *3/*3 homozygotes (4.3%, 4/92), whereas none occurred in *1 carriers (0/13).

This pattern aligns with the clinical-confounding interpretation introduced above: CYP3A5 *1 carriers in our cohort were older and had poorer renal function, and accordingly experienced clinically detectable but largely non-severe bleeding events. By contrast, major bleeding was driven predominantly by advanced age and impaired renal function—clinical factors that occurred across both genotype groups and were not concentrated in *1 carriers despite their renal disadvantage. Severity-stratified univariable and multivariable analyses confirming this dissociation are provided in Tables S3 and S4 and Figure S1 of the Supplementary Materials.

Taken together, no genotype was associated with clinically major (BARC 3/5) bleeding in this cohort: all four major events occurred in CYP3A5 *3/*3 homozygotes, and none in *1 carriers, and the corresponding odds ratio was non-significant (OR 0.73, *p* = 0.83).

### 3.7. Sensitivity and Additional Analyses

A small subset of patients (*n* = 6) had markedly elevated admission C-reactive protein levels (>50 mg/L), consistent with systemic inflammation. To assess whether these outliers influenced the primary genetic association, the primary analysis of CYP3A5 *1 carrier status was repeated after their exclusion (*n* = 99 remaining patients, 12 bleeding events). The association between CYP3A5 *1 carrier status and any bleeding remained essentially unchanged (Fisher exact OR 4.33, 95% CI 1.09–17.30; *p* = 0.049; Firth-penalized OR 4.37, 95% CI 1.09–16.30; *p* = 0.038), indicating that the observed association is not driven by this small subset of patients with marked systemic inflammation.

To address the possibility that the observed associations reflected access-site rather than spontaneous bleeding, we repeated the analysis separately for the five access-site and eight spontaneous (non-access-site) bleeding events (Table S5). For spontaneous bleeding, the clinical predictors persisted and strengthened (age ≥ 75 years OR 7.52, 95% CI 1.72–33.31, *p* = 0.008; prior chronic kidney disease OR 6.70, 95% CI 1.36–30.40, *p* = 0.022; eGFR < 60 mL/min/1.73 m^2^ OR 3.48, *p* = 0.078), whereas the corresponding associations for access-site bleeding alone were weaker and non-significant (e.g., age ≥ 75 years OR 4.63, *p* = 0.101). CYP3A5 *1 carrier status remained univariably associated with spontaneous bleeding (OR 5.30, 95% CI 1.10–23.25, *p* = 0.039), consistent with the older and more renally impaired profile of carriers. These analyses indicate that the bleeding determinants identified in this cohort are not an artifact of femoral access.

Additional exploratory analyses—including BARC severity-stratified univariable and multivariable Firth regression models (Tables S3 and S4, Figure S1) and a gradient-boosted machine-learning analysis with SHAP-based feature interpretation (Figures S2–S4)—are presented in the Supplementary Materials. These analyses confirmed (i) that the apparent association between CYP3A5 *1 carrier status and bleeding was driven predominantly by minor (BARC 1/2) events, whereas major (BARC 3/5) bleeding was dominated by advanced age and impaired renal function, and (ii) that eGFR and age were the dominant features driving predicted bleeding risk in the machine-learning model.

## 4. Discussion

Ticagrelor reduces ischemic events in ACS more reliably than clopidogrel, but at the cost of a measurable, dose-independent increase in bleeding [3,4]. The bleeding burden is not evenly distributed across patients, and most of the variability clinicians observe at the bedside is explained by old, unsexy factors: age, kidney function, anemia, prior bleeding, and body weight. Pharmacogenetic factors enter the conversation because ticagrelor relies almost entirely on CYP3A4 and, to a lesser extent, CYP3A5, for conversion to its active metabolite AR-C124910XX [8]. Reduced enzymatic activity should, in principle, raise circulating drug levels and intensify platelet inhibition; whether this translates into more bleeding has been the subject of a small, contradictory literature. The present study contributes to that literature with a single-center Serbian cohort and produces a fairly unambiguous picture: clinical factors carried almost the entire signal, and the only polymorphism with a statistically significant association, on closer inspection, was tracking the same clinical factors rather than acting independently.

In our univariable analysis, patients aged seventy-five years or older were roughly eight times more likely to bleed than younger patients, and this association persisted after mutual adjustment for renal function and genotype. eGFR below 60 mL/min/1.73 m^2^ and prior chronic kidney disease were associated with the expected magnitude and direction, with overlapping confidence intervals and partial loss of significance after adjustment for age. None of this is novel. It maps onto the variables that constitute the PRECISE-DAPT score [5] and the BARC-derived risk algorithms developed for European and Asian DAPT cohorts [6]; it also reproduces the findings of the PLATO investigators when they stratified their 18,624 patients by age and renal function [4]. The clinical interpretation is straightforward: a seventy-eight-year-old patient with an eGFR of 45 mL/min/1.73 m^2^ will likely bleed on ticagrelor regardless of which CYP3A haplotype she carries.

What is worth emphasizing is how completely these two factors absorbed the predictive signal in our cohort. In the parsimonious multivariable Firth model, age ≥ 75 years retained an adjusted odds ratio of 4.82 (*p* = 0.026), while eGFR < 60 attenuated to *p* = 0.349–a familiar pattern reflecting the strong correlation between advanced age and declining glomerular filtration. The implication, both for clinical practice and for the design of bleeding-risk scores, is that adding renal function to age yields diminishing returns once the age cut-off is incorporated. This is also why the eight-feature gradient-boosted model in our supplementary analysis achieved a respectable mean AUC of 0.704 with no help from genotype at all, and why adding CYP3A5 to that model failed to improve discrimination by even a single decimal place.

Univariable analysis identified CYP3A5 *1 carriers as having roughly fourfold higher odds of bleeding (Firth OR 4.16, *p* = 0.010). Taken in isolation, this would support a pharmacogenetic interpretation in which CYP3A5 expressors metabolize ticagrelor faster, a pattern that would predict the opposite. CYP3A5 *1 is the functional expressor allele, and CYP3A5 *3 introduces a splicing defect that abolishes the enzyme [13]. Faster ticagrelor clearance in *1 carriers should result in lower plasma concentrations, weaker platelet inhibition, and—if anything—fewer bleeding events. Our raw data points in the opposite direction. This is a problem only if the signal is causal.

The pre-specified comparison of baseline characteristics across CYP3A5 genotype groups provided the explanation. In our cohort, *1 carriers were noticeably older (33% versus 12% aged ≥75 years; *p* = 0.089), had a higher prevalence of documented chronic kidney disease (31% versus 8%; *p* = 0.029), and were more likely to be in the eGFR < 60 mL/min/1.73 m^2^ band (62% versus 29%; *p* = 0.029). This is the kind of baseline imbalance that ought not to exist if 105 consecutive patients had been allocated to genotypes by a fair coin, but with only thirteen *1 carriers, chance can produce striking skews. As a result, the unadjusted odds ratio for CYP3A5 *1 carrier status reflects, in part, older age and poor kidney function.

After accounting for age and renal function, the CYP3A5 *1 association did not survive adjustment-adjusted OR 3.31 (95% CI 0.71–14.72; *p* = 0.123) in the continuous-predictor model and 2.57 (95% CI 0.55–10.67; *p* = 0.220) in the binary parsimonious model, both intervals comfortably including the null. This is what one expects if the univariate signal is confounded rather than causal: once the age and renal-function imbalance between genotype groups is accounted for, no independent CYP3A5 effect remains. The gradient-boosted analysis in our Supplementary Materials—which is not constrained by the linearity assumption—agrees, finding that CYP3A5 carried essentially no additional predictive information beyond what age and eGFR already supplied. The most defensible reading is that CYP3A5 *1 carrier status is a partial proxy for advanced age and impaired renal function in this cohort.

CYP3A5 *1 carriers experienced minor bleeding (BARC 1/2) at 31%, compared with 5% in *3/*3 homozygotes. None of the four major bleeding events (BARC 3/5) occurred in *1 carriers; all four occurred in *3/*3 homozygotes, and all were 65 years or older, with two in the ≥75 group. The minor-bleeding signal was concentrated in the genotype group that was both older and more renally impaired—exactly where minor bleeding is expected to accumulate on clinical grounds, and exactly where ticagrelor’s pharmacokinetic forgiveness narrows [7]. Major bleeding, the outcome that actually matters for patient management, was not associated with genotype at all. The clinical implication is that, in this cohort, knowing a patient’s CYP3A5 status would have added little to a risk assessment that already accounted for her age, eGFR, and a brief review of the chart.

Earlier pharmacokinetic studies in healthy Chinese subjects found no effect of CYP3A5 *3 on ticagrelor exposure or platelet inhibition [14]. Holmberg and colleagues, who detected a CYP3A4*22 effect in their Finnish volunteers, found no CYP3A5 effect even in a carefully controlled crossover design [10]. The POPular Genetics sub-study by Azzahhafi and colleagues—by far the largest ticagrelor pharmacogenetic dataset to date, with 1281 STEMI patients—likewise reported no association of CYP3A5 expressor status with either the bleeding endpoint (adjusted HR 0.97 for major and minor PLATO bleeding) or the thrombotic endpoint [12]. Our apparently positive univariable finding fits into this picture as an artifact of the small, imbalanced cohort rather than as a correction to it.

The strongest a priori candidate for a pharmacogenetic effect on ticagrelor-related bleeding is CYP3A4*22 (rs35599367, c.522-191C>T). The T-allele lies in intron 6 of CYP3A4 and reduces hepatic CYP3A4 mRNA expression and enzyme activity by roughly half [9]. In a controlled pharmacokinetic study comparing seven *22 heterozygotes with eight non-carriers, Holmberg and colleagues observed a 60% increase in ticagrelor AUC and nearly a doubling of platelet inhibition among carriers [10]. In a Finnish biobank study of 368 patients with ischemic heart disease on ticagrelor, Liedes and colleagues subsequently reported a hazard ratio of 3.74 (95% CI 1.26–11.05) for bleeding among *22 carriers, an effect that persisted after adjustment for age, sex, and aspirin co-medication [11]. Before the present analysis, this was the genotype we expected to show an effect.

Eight of our 105 patients carried at least one *22 allele (none was homozygous); one of these eight bled. The unadjusted Firth odds ratio of 1.37, with a *p*-value of 0.109, is statistically non-significant, and the 95% confidence interval (0.19–9.64) is so wide that the data are formally compatible with anything from a tenfold protective effect to a tenfold risk. Our cohort simply does not have the power to settle the question. With a *22 minor allele frequency of 3.81—close to the Finnish estimate of 3.0% [11] and the Central European value of around 5% [29]—and a bleeding incidence of 12.4%, the expected number of bleeds in *22 carriers, if the Liedes hazard ratio held, would be slightly under two; observing one is entirely consistent with that point estimate, and observing zero would have been too.

The same null applies in the POPular Genetics sub-study, where 152 CYP3A4*22 carriers among 1281 ticagrelor-treated STEMI patients yielded an adjusted hazard ratio of 0.93 (95% CI 0.58–1.50) for the composite of PLATO major and minor bleeding [12]. That study had greater statistical power than ours and failed to confirm Liedes’s signal. The CYP3A4*22 story is therefore not settled. Three reasonably designed studies—Holmberg in volunteers [10], Liedes in a registry cohort [11], and Azzahhafi in a randomized trial sub-study [12]—give three different answers, and the divergence is likely due to differences in how exposure was defined, how patients were selected, and how bleeding was adjudicated. Our contribution here is to add another zero to that literature: no signal, very wide intervals, and statistical power consistent with those intervals. We would not draw a conclusion either way from our eight *22 carriers.

The mechanistic case for a CYP3A4*22 effect on bleeding remains plausible. The Holmberg pharmacokinetic data are clean, the molecular basis of *22 is reasonably understood, and ticagrelor’s dependence on CYP3A4 is established [8]. What remains uncertain is whether the magnitude of the pharmacokinetic effect—a 60% AUC increase in *22 heterozygotes [10] versus a typical inter-individual coefficient of variation of 30–50% for ticagrelor [7]—is large enough to produce a clinically detectable shift in bleeding rates in the real-world setting, where many other factors operate at a similar magnitude. Larger, adequately powered, prospective studies that genotype before treatment initiation are the only way to resolve this; pooled analyses of existing cohorts may help in the interim.

CYP4F2 is a fatty-acid omega-hydroxylase involved in the synthesis of 20-HETE [15]. Its link to ticagrelor is indirect at best. The rs3093135 variant is an intronic A>T polymorphism in CYP4F2, with the T allele as the minor allele. Early mechanistic models suggested that 20-HETE may inhibit platelet aggregation by antagonizing thromboxane receptor signaling [18]; however, more recent evidence indicates that 20-HETE-mediated platelet effects are complex and may involve downstream lipid metabolites rather than direct receptor antagonism [16,17]. The original Lithuanian report by Tatarunas and colleagues, comparing clopidogrel- and ticagrelor-treated patients, found that T-allele carriers had lower platelet aggregation values [18]; a later analysis from the same group, with overlapping patients, instead reported increased odds of bleeding in T-allele carriers [19]. Liedes and colleagues found no signal in their Finnish biobank cohort [11]. Our results are consistent with the Finnish null. The Firth odds ratio for CYP4F2 T-carrier status versus any bleeding was 1.17 (95% CI 0.38–3.67; *p* = 0.111), and the recessive comparison (T/T versus the rest) was likewise non-significant. Whatever signal Tatarunas observed is not present in our patients, and the same caveat as for *22 applies—with 39 T-allele carriers and six homozygotes, we have limited power to detect moderate-sized effects.

It is also worth situating these findings within the current landscape of antiplatelet pharmacogenetics. Guideline-endorsed implementation to date centers almost entirely on CYP2C19-guided selection or de-escalation of clopidogrel; no comparable, clinically validated pathway exists for CYP3A-guided ticagrelor prescribing, and our null and confounded findings are concordant with that state of evidence. This matters clinically because bleeding during the index admission is not prognostically neutral: in-hospital bleeding in ACS is associated with adverse modification of post-discharge therapy and worse mid-term prognosis [30], which is precisely why accurate, parsimonious bleeding-risk stratification is valuable.

We relied on Firth’s penalized maximum likelihood throughout rather than ordinary logistic regression [25,26]. In our primary multivariable model, with 13 bleeding events and an events-per-predictor ratio of four, ordinary maximum-likelihood estimates would be biased away from the null, and the confidence intervals would be unreliable. Firth’s penalty, based on Jeffreys’s invariant prior, removes the first-order asymptotic bias of the MLE and, importantly, yields finite estimates and intervals even when complete or quasi-complete separation occurs—a real risk in our BARC severity-stratified analyses, where the *1 carrier group contained zero BARC 3/5 events. Penalized likelihood-ratio tests, rather than Wald tests, were used for inference on individual coefficients [27]. This is the conventional approach to sparse-event regression in the medical literature; however, it does not increase our cohort size.

Second, the gradient-boosted machine-learning analysis in the Supplementary Materials is intentionally subordinate. Out-of-fold AUCs of 0.63 to 0.70 are not impressive in absolute terms—a well-calibrated PRECISE-DAPT score reaches roughly 0.70 to 0.72 in external validation [5]—and the principal value of the XGBoost analysis is not its predictive accuracy but the consistency of its variable importances with the regression results. SHAP analysis ranked eGFR and age above CYP3A5; the discriminative gain from adding CYP3A5 to the clinical model was indistinguishable from zero (mean ΔAUC = +0.001 in the parsimonious model; −0.003 in the broader eight-feature model). When a non-linear model with no stake in the regression’s parametric assumptions arrives at the same conclusion as the parametric model, this is at least a partial check on the robustness of the interpretation. It is not a substitute for replication in a larger sample.

The principal strength of this work is the methodological rigor applied to data that are often analyzed less carefully. Hardy–Weinberg equilibrium was tested using both Pearson’s chi-square and the exact procedure of Wigginton and colleagues for each of the three polymorphisms [24]; all three were in equilibrium, and the observed minor allele frequencies were consistent with published European reference values. Firth-penalized regression was used throughout rather than ordinary maximum likelihood, and a pre-specified sensitivity analysis excluded patients with admission C-reactive protein above 50 mg/L without altering any of the principal conclusions. Genotype-stratified baseline analysis was performed before multivariable adjustment, and the resulting imbalance was reported transparently rather than concealed. Clinical phenotyping was complete: every patient was treated at the same center by the same interventional and clinical team, every patient received aspirin, a statin, and a proton-pump inhibitor, and bleeding events were classified according to BARC criteria [22] by a clinical investigator who reviewed the source records.

The cohort of 105 patients with 13 bleeding events is too small to detect anything other than very large genetic effects, and the absence of a CYP3A4*22 signal cannot distinguish a true null from a type-II error. Multiplicative interactions among CYP3A4, CYP3A5, and CYP4F2—the kind of epistatic effects that may matter for a drug with multiple metabolic routes—are essentially untestable at this sample size, and we did not attempt to test them. The single-center design limits generalizability beyond Niš and Serbia; haplotype frequencies in our cohort were comparable to European reference data, but the clinical case mix reflects regional practice patterns. We did not measure ticagrelor plasma concentrations or platelet aggregation, so any inference about a pharmacokinetic or pharmacodynamic mechanism is necessarily indirect. Follow-up was six months; some delayed bleeding events that occur during prolonged DAPT would have been missed.

With 13 bleeding events and only four major events, the study is powered to detect only very large genetic effects, and the wide confidence intervals around the genetic estimates directly reflect this; a true null cannot be distinguished from a type-II error for CYP3A4*22 or CYP4F2. The single-center design and the high proportion of femoral access (96.2%) further limit generalizability to contemporary, predominantly radial ACS practice. Multicenter replication in genetically distinct, real-world cohorts will be required before any clinical implementation can be considered.

The study was designed around bleeding as the primary safety outcome, and ischemic and thrombotic efficacy endpoints were not pre-specified as analytic outcomes. Only a small number of recurrent ischemic or thrombotic events and deaths occurred during the six-month follow-up—too few to support any genotype-stratified efficacy analysis. As a result, the net clinical benefit of ticagrelor in this cohort—the balance between bleeding and ischemic risk, the metric of ultimate clinical interest—cannot be assessed, which is an important limitation. Whether the variants studied here shift that balance in either direction will require larger, adequately powered, genotype-stratified studies that capture both bleeding and ischemic endpoints.

Multiple clinical and genetic predictors were evaluated without correction for multiple comparisons; the analyses are therefore exploratory, and the single nominally significant genetic association in the cohort, univariable CYP3A5 *1 carrier status (*p* = 0.043), would not survive correction for the number of predictors tested and should be regarded as hypothesis-generating.

One additional consideration warrants mention. The CYP3A4*1G variant (rs2242480) was genotyped in this cohort but is omitted from the present analysis. Available evidence on its functional impact on CYP3A4 activity and expression is inconsistent across studies, and the PharmVar Consortium has cautioned against classifying it as a robust functional star allele because the variant occurs on multiple haplotype backgrounds and no clinically reproducible effect has been demonstrated to date. We considered it more appropriate to exclude it from a clinically oriented analysis than to report results for a marker whose functional status is under active reconsideration. The CYP3A4*1G genotypes obtained in this cohort will be reported separately as part of a doctoral thesis project.

Three implications follow from this work, and we will state them plainly. First, in the routine clinical setting, we study—Serbian patients with ACS undergoing PCI or medical management after coronary angiography and receiving ticagrelor-based DAPT—the variables that predict bleeding are the same as those that have always predicted it. Age above 75 years and an eGFR below 60 mL/min/1.73 m^2^ together identify a subgroup whose in-hospital and six-month bleeding risk is several times the cohort average, and this remains true after adjusting for genotype. Clinicians deciding between ticagrelor and clopidogrel, or between standard and shortened DAPT duration, will derive most of the available benefit by attending to these two variables; the PRECISE-DAPT score formalizes this attention, and we would encourage its use.

Second, based on this study’s evidence, the genotypes we tested do not provide additional information beyond what age and renal function already offer. We are explicit that this is a statement about predictive utility in a 105-patient, single-center cohort, not a definitive statement about underlying biology. CYP3A4*22, in particular, has a plausible pharmacokinetic mechanism and a positive registry-based study; the case is not closed. We conclude that preemptive genotyping for these three variants is unlikely to change patient management in our setting, and that supporting evidence for any such recommendation in the broader literature remains weak and contradictory.

Third, future studies in this area would benefit from larger sample sizes, pretreatment genotyping with confirmed exposure, measurement of plasma concentrations of ticagrelor and AR-C124910XX, and explicit handling of the confounding between CYP3A5 genotype and renal function that we and others have observed. Multi-center pooling of existing cohorts is one approach; a prospective genotype-stratified outcome study is another. The POPular Genetics sub-study [12] sets the benchmark for such a study, and our cohort is best understood as a small Serbian contribution to the larger evidence base. Whether CYP3A4*22 will ultimately prove a useful clinical marker for ticagrelor remains an open empirical question; the present data do not resolve it.

## 5. Conclusions

In this single-center observational study of 105 Serbian ACS patients receiving ticagrelor-based DAPT, the primary predictors of bleeding at six months were variables clinicians already address in routine practice: age, particularly age ≥ 75 years, and impaired renal function, expressed either as a continuous decline in eGFR or as the conventional 60 mL/min/1.73 m^2^ threshold. These two factors accounted for nearly the entire predictive signal in our cohort and remained independent in penalized multivariable models.

None of the three CYP polymorphisms we tested provided clinically useful information beyond age and renal function. The CYP3A5 *1 univariable signal (OR 4.16, *p* = 0.043) did not survive adjustment for age and renal function (adjusted *p* = 0.12 and 0.22), reflecting the older and more renally impaired profile of carriers; what little signal remained was confined to minor (BARC 1/2) bleeding, and no genotype was associated with major (BARC 3/5) bleeding. CYP3A4*22—the variant with the strongest prior biological rationale and the most discussed in the recent ticagrelor pharmacogenetic literature—produced a numerically positive but statistically inert estimate with wide confidence intervals, consistent with the substantially larger POPular Genetics sub-study, which failed to replicate the earlier Finnish register signal. CYP4F2 rs3093135 did not change.

Based on this evidence, preemptive genotyping of CYP3A4*22, CYP3A5*3, and CYP4F2 rs3093135 is unlikely to improve bleeding-risk assessment in this patient population beyond what age and renal function already provide. Whether CYP3A4*22 will prove clinically actionable in a larger, prospective, genotype-stratified study with measured plasma concentrations of ticagrelor and AR-C124910XX remains an open question. The present results do not resolve it; they place a small Serbian contribution on the null side of the existing literature and suggest that the clinical instinct to focus on the patient in front of you—her age, her kidneys, her bleeding history—remains more useful than her genotype.

## Figures and Tables

**Table 1:** Baseline demographic, clinical, laboratory, procedural, and medication characteristics of the study cohort, presented overall and stratified by any bleeding event. Continuous variables are reported as medians (interquartile ranges) and compared using the Mann–Whitney U test. Categorical variables are reported as counts (percentages) and compared using the Fisher exact test for binary variables or the chi-square test for multilevel categorical variables. Abbreviations: ACS, acute coronary syndrome; ALT, alanine aminotransferase; AST, aspartate aminotransferase; BMI, body mass index; CCB, calcium-channel blocker; CRP, C-reactive protein; eGFR, estimated glomerular filtration rate; HDL-C, high-density lipoprotein cholesterol; Hct, hematocrit; Hgb, hemoglobin; LDL-C, low-density lipoprotein cholesterol; LVEF, left ventricular ejection fraction; MRA, mineralocorticoid receptor antagonist; NSTEMI, non-ST-segment elevation myocardial infarction; PCI, percutaneous coronary intervention; PPI, proton-pump inhibitor; SGLT2, sodium–glucose co-transporter 2; STEMI, ST-segment elevation myocardial infarction; UA, unstable angina; WBC, white blood cell count.

Variable	Overall (*N* = 105)	No Bleeding (*n* = 92)	Bleeding (*n* = 13)	*p* Value
Age, years	64.0 (54.0–71.0)	63.0 (53.8–68.0)	71.0 (67.0–81.0)	0.002
Age ≥ 75 years	15 (14.3%)	9 (9.8%)	6 (46.2%)	0.003
Male sex	79 (75.2%)	70 (76.1%)	9 (69.2%)	0.732
BMI, kg/m^2^	25.9 (24.7–27.5)	25.9 (24.7–27.5)	26.8 (25.0–27.5)	0.722
Weight, kg	85.0 (76.0–90.0)	85.5 (76.0–90.2)	80.0 (75.0–85.0)	0.124
Current smoker	37 (35.2%)	34 (37.0%)	3 (23.1%)	0.536
Hypertension	89 (84.8%)	78 (84.8%)	11 (84.6%)	1.000
Diabetes mellitus type 2	40 (38.1%)	36 (39.1%)	4 (30.8%)	0.762
Prior CKD	11 (10.5%)	7 (7.6%)	4 (30.8%)	0.029
Prior bleeding	1 (1.0%)	0 (0.0%)	1 (7.7%)	0.124
Prior thrombosis	4 (3.8%)	4 (4.3%)	0 (0.0%)	1.000
Prior CAD	30 (28.6%)	25 (27.2%)	5 (38.5%)	0.512
ACS type				0.499
UA	9 (8.6%)	9 (9.8%)	0 (0.0%)	
STEMI	74 (70.5%)	64 (69.6%)	10 (76.9%)	
NSTEMI	22 (21.0%)	19 (20.7%)	3 (23.1%)	
Killip class				1.000
I	102 (97.1%)	89 (96.7%)	13 (100.0%)	
II	3 (2.9%)	3 (3.3%)	0 (0.0%)	
LVEF, %	48.0 (44.0–54.0)	48.0 (44.8–53.0)	52.0 (40.0–55.0)	0.770
Hgb at admission, g/L	144.0 (134.0–153.0)	144.0 (136.0–153.0)	132.0 (128.0–163.0)	0.368
Hct, %	41.0 (38.0–44.0)	41.0 (38.0–44.0)	37.0 (36.0–46.0)	0.230
WBC, ×10^9^/L	10.8 (8.8–13.1)	10.9 (8.8–13.1)	9.7 (8.9–11.4)	0.319
Platelets, ×10^9^/L	242.0 (194.0–283.0)	242.5 (194.0–283.2)	211.0 (196.0–255.0)	0.508
CRP, mg/L	3.9 (2.0–9.6)	3.9 (2.2–8.2)	3.6 (2.0–24.1)	0.550
Glucose, mmol/L	7.2 (6.2–9.4)	7.2 (6.2–9.4)	7.3 (6.4–8.9)	0.922
Creatinine, μmol/L	88.2 (79.9–106.5)	87.9 (79.9–101.1)	108.8 (85.3–126.0)	0.060
Urea, mmol/L	6.5 (5.2–7.9)	6.3 (4.9–7.7)	7.6 (6.0–10.0)	0.047
eGFR, mL/min/1.73 m^2^	71.0 (54.0–81.7)	72.5 (57.1–82.0)	49.6 (43.8–67.1)	0.019
eGFR < 60 mL/min/1.73 m^2^	35 (33.3%)	27 (29.3%)	8 (61.5%)	0.029
AST, U/L	32.0 (23.0–66.0)	31.0 (23.0–63.8)	35.0 (28.0–70.0)	0.613
ALT, U/L	29.0 (21.0–43.0)	28.5 (21.0–42.2)	31.0 (22.0–48.0)	0.493
Troponin, peak	1.0 (0.2–7.7)	0.9 (0.2–7.3)	2.8 (0.3–7.9)	0.340
Total cholesterol, mmol/L	5.2 (4.3–6.3)	5.2 (4.4–6.4)	3.9 (3.6–5.8)	0.172
LDL-C, mmol/L	3.1 (2.2–4.0)	3.1 (2.5–4.0)	2.1 (1.2–3.6)	0.066
HDL-C, mmol/L	1.2 (1.0–1.4)	1.2 (1.0–1.4)	1.0 (1.0–1.4)	0.474
Triglycerides, mmol/L	1.9 (1.4–2.5)	1.9 (1.4–2.5)	1.4 (1.1–2.1)	0.201
PCI with stent implantation	99 (94.3%)	87 (94.6%)	12 (92.3%)	0.557
Access site				1.000
Femoral	101 (96.2%)	88 (95.7%)	13 (100.0%)	
Radial	4 (3.8%)	4 (4.3%)	0 (0.0%)	
Number of stents	1.0 (1.0–2.0)	1.0 (1.0–2.0)	1.0 (1.0–2.0)	0.708
Number of affected vessels	1.0 (1.0–2.0)	1.0 (1.0–2.0)	1.0 (1.0–1.0)	0.390
Statin	105 (100.0%)	92 (100.0%)	13 (100.0%)	–
PPI	105 (100.0%)	92 (100.0%)	13 (100.0%)	–
β-blocker	89 (84.8%)	78 (84.8%)	11 (84.6%)	1.000
ACE inhibitor	76 (72.4%)	68 (73.9%)	8 (61.5%)	0.341
MRA	43 (41.0%)	36 (39.1%)	7 (53.8%)	0.373
SGLT2 inhibitor	53 (50.5%)	45 (48.9%)	8 (61.5%)	0.555
CCB	12 (11.4%)	12 (13.0%)	0 (0.0%)	0.354
Length of stay, days	4.0 (3.0–5.0)	4.0 (3.0–5.0)	7.0 (5.0–10.0)	<0.001

**Table 2:** Genotype distribution, minor allele frequencies (MAF), and Hardy–Weinberg equilibrium (HWE) test results for the three CYP polymorphisms examined. Exact HWE *p* values are reported using the method of Wigginton, Cutler, and Abecasis (2005) [24]. All *p*-values > 0.05 indicate no significant deviation from HWE.

SNP	Genotype 1	Genotype 2	Genotype 3	MAF	HWE *p*
CYP3A4*22 rs35599367	CC: 97 (92.4%)	CT: 8 (7.6%)	TT: 0 (0.0%)	3.81%	1.000
CYP3A5 rs776746	*3/*3: 92 (87.6%)	*1/*3: 13 (12.4%)	*1/*1: 0 (0.0%)	6.19%	1.000
CYP4F2 rs3093135	AA: 60 (57.1%)	AT: 39 (37.1%)	TT: 6 (5.7%)	24.29%	1.000

**Table 3:** Univariable associations between principal clinical and genetic predictors and any bleeding, estimated using Firth’s penalized logistic regression. Odds ratios (OR), 95% confidence intervals (CI), and penalized likelihood-ratio test (LRT) *p* values are reported. For continuous variables, effects are expressed per unit increase. Genotype effects are modeled under the dominant model. The complete univariable analysis, including all evaluated predictors, is provided in Table S1.

Predictor	OR (95% CI)	*p*
Age (per 1 year)	1.12 (1.04–1.21)	0.001
Age ≥ 75 years	7.62 (2.17–27.15)	0.002
Male sex	0.67 (0.21–2.49)	0.532
Hypertension	0.85 (0.22–4.72)	0.831
Diabetes mellitus type 2	0.73 (0.20–2.33)	0.607
Prior CKD	5.40 (1.32–20.69)	0.020
Prior CAD	1.71 (0.51–5.42)	0.373
eGFR (per 1 mL/min/1.73 m^2^)	0.96 (0.93–0.99)	0.012
eGFR < 60 mL/min/1.73 m^2^	3.68 (1.17–12.48)	0.026
Hgb at admission (per 1 g/L)	0.99 (0.95–1.03)	0.479
CRP (per 1 mg/L)	1.02 (0.99–1.05)	0.139
CYP3A4*22 carrier (vs. CC)	1.37 (0.14–7.11)	0.747
CYP3A5 *1 carrier (vs. *3/*3)	4.16 (1.05–15.13)	0.043
CYP4F2 T carrier (vs. AA)	1.17 (0.37–3.66)	0.781

**Table 4:** Multivariable Firth’s penalized logistic regression models for any bleeding. Model A includes age and eGFR as continuous predictors, along with prior chronic kidney disease and CYP3A5 *1 carrier status. Model B (parsimonious model) uses clinically interpretable binary cut-points (age ≥ 75 years and eGFR < 60 mL/min/1.73 m^2^), along with CYP3A5 *1 carrier status. Adjusted odds ratios (OR), 95% confidence intervals (CI), and penalized likelihood-ratio test (LRT) *p* values are reported for each predictor.

Predictor	Adjusted OR (95% CI)	*p*
Model A—continuous predictors		
Age (per yr)	1.09 (1.01–1.19)	0.019
eGFR (per mL/min/1.73 m^2^)	0.99 (0.95–1.03)	0.667
Prior CKD	1.51 (0.25–7.90)	0.639
CYP3A5 *1 carrier	3.31 (0.71–14.72)	0.123
Model B—binary cut-points (parsimonious)		
Age ≥ 75 years	4.82 (1.21–18.98)	0.026
eGFR < 60 mL/min/1.73 m^2^	1.89 (0.48–7.22)	0.349
CYP3A5 *1 carrier	2.57 (0.55–10.67)	0.220

**Table 5:** Unadjusted bleeding outcomes, stratified by BARC severity and by CYP3A5 rs776746 genotype. Event counts are shown as *n*/total (percentage). Odds ratios with 95% confidence intervals were calculated using the Haldane-corrected formula, and *p* values were calculated using Fisher’s exact test. BARC 1/2 (minor) bleeding includes events not requiring intervention; BARC 3/5 (major) bleeding includes events requiring intervention, transfusion, or causing death.

Outcome	CYP3A5 *3/*3 (*n* = 92)	CYP3A5 *1 Carrier (*n* = 13)	OR (95% CI)	*p* Value
In-hospital bleeding (any)	7/92 (7.6%)	4/13 (30.8%)	5.40 (1.32–22.05)	0.029
BARC 1/2 (minor)	5/92 (5.4%)	4/13 (30.8%)	7.73 (1.75–34.08)	0.013
BARC 3/5 (major)	4/92 (4.3%)	0/13 (0.0%)	0.73 (0.04–14.30)	1.000
Any bleeding (composite)	9/92 (9.8%)	4/13 (30.8%)	4.10 (1.05–16.03)	0.054
Bleeding within 6 months	2/92 (2.2%)	0/13 (0.0%)	1.34 (0.06–29.46)	1.000

## Data Availability

The anonymized dataset that supports the findings of this study is available from the corresponding author upon reasonable request, subject to compliance with the Serbian Personal Data Protection Act and applicable institutional policies. Genetic data have been pseudonymized and are stored on encrypted institutional servers.

## Supplementary Materials

The following supporting information can be downloaded at: https://www.mdpi.com/article/10.3390/medicina62061202/s1.

## References

[1] Tsao C.W., Aday A.W., Almarzooq Z.I., Anderson C.A.M., Arora P., Avery C.L., Baker-Smith C.M., Beaton A.Z., Boehme A.K., Buxton A.E. (2023). Heart Disease and Stroke Statistics–2023 Update: A Report from the American Heart Association. Circulation.

[2] Byrne R.A., Rossello X., Coughlan J.J., Barbato E., Berry C., Chieffo A., Claeys M.J., Dan G.-A., Dweck M.R., Galbraith M. (2024). 2023 ESC Guidelines for the Management of Acute Coronary Syndromes. Eur. Heart J..

[3] Dobesh P.P., Oestreich J.H. (2014). Ticagrelor: Pharmacokinetics, Pharmacodynamics, Clinical Efficacy, and Safety. Pharmacotherapy.

[4] Wallentin L., Becker R.C., Budaj A., Cannon C.P., Emanuelsson H., Held C., Horrow J., Husted S., James S., Katus H. (2009). Ticagrelor versus Clopidogrel in Patients with Acute Coronary Syndromes. N. Engl. J. Med..

[5] Costa F., van Klaveren D., James S., Heg D., Räber L., Feres F., Pilgrim T., Hong M.K., Kim H.S., Colombo A. (2017). Derivation and Validation of the Predicting Bleeding Complications in Patients Undergoing Stent Implantation and Subsequent Dual Antiplatelet Therapy (PRECISE-DAPT) Score: A Pooled Analysis of Individual-Patient Datasets from Clinical Trials. Lancet.

[6] Urban P., Mehran R., Colleran R., Angiolillo D.J., Byrne R.A., Capodanno D., Cuisset T., Cutlip D., Eerdmans P., Eikelboom J. (2019). Defining High Bleeding Risk in Patients Undergoing Percutaneous Coronary Intervention. Circulation.

[7] Teng R. (2015). Ticagrelor: Pharmacokinetic, Pharmacodynamic and Pharmacogenetic Profile: An Update. Clin. Pharmacokinet..

[8] Zhou D., Andersson T.B., Grimm S.W. (2011). In Vitro Evaluation of Potential Drug–Drug Interactions with Ticagrelor: Cytochrome P450 Reaction Phenotyping, Inhibition, Induction, and Differential Kinetics. Drug Metab. Dispos..

[9] Wang D., Guo Y., Wrighton S.A., Cooke G.E., Sadee W. (2011). Intronic Polymorphism in CYP3A4 Affects Hepatic Expression and Response to Statin Drugs. Pharmacogenom. J..

[10] Holmberg M.T., Tornio A., Paile-Hyvärinen M., Tarkiainen E.K., Neuvonen M., Neuvonen P.J., Backman J.T., Niemi M. (2019). CYP3A4*22 Impairs the Elimination of Ticagrelor, but Has No Significant Effect on the Bioactivation of Clopidogrel or Prasugrel. Clin. Pharmacol. Ther..

[11] Liedes H., Pajula J., Vuorinen A.-L., De Pretis F., van Gils M., Harno K., Lehto M., Niemi M., Lähteenmäki J. (2023). CYP3A4*22 May Increase Bleeding Risk in Ticagrelor Users. Basic Clin. Pharmacol. Toxicol..

[12] Azzahhafi J., Bergmeijer T.O., van den Broek W.W.A., Chan Pin Yin D.R.P.P., Rayhi S., Peper J., Bor W.L., Claassens D.M.F., van Schaik R.H.N., ten Berg J.M. (2022). Effects of CYP3A4*22 and CYP3A5 on Clinical Outcome in Patients Treated with Ticagrelor for ST-Segment Elevation Myocardial Infarction: POPular Genetics Sub-Study. Front. Pharmacol..

[13] Kuehl P., Zhang J., Lin Y., Lamba J., Assem M., Schuetz J., Watkins P.B., Daly A., Wrighton S.A., Hall S.D. (2001). Sequence Diversity in CYP3A Promoters and Characterization of the Genetic Basis of Polymorphic CYP3A5 Expression. Nat. Genet..

[14] Li M., Hu Y., Li H., Wen Z., Hu X., Zhang D., Zhang Y., Xiao J., Tang J., Chen X. (2017). No Effect of SLCO1B1 and CYP3A4/5 Polymorphisms on the Pharmacokinetics and Pharmacodynamics of Ticagrelor in Healthy Chinese Male Subjects. Biol. Pharm. Bull..

[15] Alvarellos M.L., Sangkuhl K., Daneshjou R., Whirl-Carrillo M., Altman R.B., Klein T.E. (2015). PharmGKB Summary: Very Important Pharmacogene Information for CYP4F2. Pharmacogenet. Genom..

[16] Tatarunas V., Jankauskiene L., Kupstyte N., Skipskis V., Gustiene O., Grybauskas P., Lesauskaite V. (2014). The Role of Clinical Parameters and of CYP2C19 G681 and CYP4F2 G1347A Polymorphisms on Platelet Reactivity during Dual Antiplatelet Therapy. Blood Coagul. Fibrinolysis.

[17] Yu J., Waresi M., Zhong H., Wu H., Ge J. (2025). 20-HETE Induced Platelet Activation via a GPR75-Independent Pathway. Thromb. Res..

[18] Tatarunas V., Kupstyte N., Zaliunas R., Giedraitiene A., Lesauskaite V. (2017). The Impact of Clinical and Genetic Factors on Ticagrelor and Clopidogrel Antiplatelet Therapy. Pharmacogenomics.

[19] Tatarunas V., Kupstyte-Kristapone N., Norvilaite R., Tamakauskas V., Skipskis V., Veikutiene A., Jakuska P., Lesauskaite V. (2019). The Impact of CYP2C19 and CYP4F2 Variants and Clinical Factors on Treatment Outcomes during Antiplatelet Therapy. Pharmacogenomics.

[20] Firth D. (1993). Bias Reduction of Maximum Likelihood Estimates. Biometrika.

[21] Levey A.S., Bosch J.P., Lewis J.B., Greene T., Rogers N., Roth D. (1999). A More Accurate Method to Estimate Glomerular Filtration Rate from Serum Creatinine: A New Prediction Equation. Modification of Diet in Renal Disease Study Group. Ann. Intern. Med..

[22] Mehran R., Rao S.V., Bhatt D.L., Gibson C.M., Caixeta A., Eikelboom J., Kaul S., Wiviott S.D., Menon V., Nikolsky E. (2011). Standardized Bleeding Definitions for Cardiovascular Clinical Trials: A Consensus Report from the Bleeding Academic Research Consortium. Circulation.

[23] Pharmacogene Variation Consortium PharmVar: Pharmacogene Variation Database. https://www.pharmvar.org.

[24] Wigginton J.E., Cutler D.J., Abecasis G.R. (2005). A Note on Exact Tests of Hardy–Weinberg Equilibrium. Am. J. Hum. Genet..

[25] Heinze G., Schemper M. (2002). A Solution to the Problem of Separation in Logistic Regression. Stat. Med..

[26] Puhr R., Heinze G., Nold M., Lusa L., Geroldinger A. (2017). Firth’s Logistic Regression with Rare Events: Accurate Effect Estimates and Predictions?. Stat. Med..

[27] van Smeden M., Moons K.G.M., de Groot J.A.H., Collins G.S., Altman D.G., Eijkemans M.J.C., Reitsma J.B. (2019). Sample Size for Binary Logistic Prediction Models: Beyond Events per Variable Criteria. Stat. Methods Med. Res..

[28] Lundberg S.M., Lee S.-I., Guyon I., Luxburg U.V., Bengio S., Wallach H., Fergus R., Vishwanathan S., Garnett R. (2017). A Unified Approach to Interpreting Model Predictions. Advances in Neural Information Processing Systems 30 (NIPS 2017).

[29] Zhou Y., Ingelman-Sundberg M., Lauschke V.M. (2017). Worldwide Distribution of Cytochrome P450 Alleles: A Meta-Analysis of Population-Scale Sequencing Projects. Clin. Pharmacol. Ther..

[30] Spadafora L., Betti M., D’Ascenzo F., De Ferrari G., De Filippo O., Gaudio C., Collet C., Sabouret P., Agostoni P., Zivelonghi C. (2025). Impact of In-Hospital Bleeding on Postdischarge Therapies and Prognosis in Acute Coronary Syndromes. J. Cardiovasc. Pharmacol..

