# Supplementary material for: CYP3A4, CYP3A5, and CYP4F2 Polymorphisms and Bleeding Risk in Ticagrelor-Based Dual Antiplatelet Therapy
Source: Medicina (Kaunas). 2026 Jun 22;62(6):1202. doi: 10.3390/medicina62061202 (PMC13304341; doi:10.3390/medicina62061202)
Supplement: Supplementary file 1 [file medicina-62-01202-s001.zip › medicina-4356807-supplementary.pdf]

Supplementary Material

This Supplementary Material includes: (i) the full univariable Firth's penalized logistic regression analysis, including all candidate predictors evaluated (Table S1); (ii) a comparison of baseline characteristics across CYP3A5 genotype groups, documenting the imbalance that prompted multivariable adjustment in the main manuscript (Table S2); (iii) univariable and multivariable Firth's regression analyses stratified by Bleeding Academic Research Consortium (BARC) severity (Tables S3–S4 and Figure S1); and (iv) a complementary machine-learning analysis using extreme gradient boosting (XGBoost) with SHAP-based feature interpretation (Figures S2–S4).

S1. Full Univariable Firth's Penalized Logistic Regression

Table S1 lists all candidate predictors evaluated with univariable Firth's penalized logistic regression for the primary composite outcome (any bleeding event). Predictors included demographics, comorbidities, admission laboratory values, and the three CYP polymorphisms of interest. The principal predictors are shown in Table 3 of the main manuscript.

Table S1. Complete univariable associations between all evaluated candidate predictors and any bleeding, estimated using Firth's penalized logistic regression. Odds ratios (OR), 95% confidence intervals (CI), and penalized likelihood-ratio test (LRT) p values are reported. For continuous variables, effects are expressed per unit increase. Genotype effects are modeled under the dominant model.

Predictor	OR (95% CI)	p value
Age (per 1 year)	1.12 (1.04–1.21)	<0.001
Age ≥ 75 years	7.62 (2.17–27.15)	0.002
Male sex	0.67 (0.21–2.49)	0.532
BMI (per 1 kg/m²)	1.03 (0.78–1.30)	0.839
Weight (per 1 kg)	0.97 (0.92–1.02)	0.201
Current smoker	0.57 (0.14–1.89)	0.366
Hypertension	0.85 (0.22–4.72)	0.831
Diabetes mellitus type 2	0.73 (0.20–2.33)	0.607
Prior CKD	5.40 (1.32–20.69)	0.020
Prior CAD	1.71 (0.51–5.42)	0.373
Hgb at admission (per 1 g/L)	0.99 (0.95–1.03)	0.479
Hct at admission (per 1%)	0.94 (0.82–1.08)	0.383
Platelets (per 10^9/L)	1.00 (0.99–1.01)	0.607
CRP (per 1 mg/L)	1.02 (0.99–1.05)	0.139
Creatinine (per 1 μmol/L)	1.02 (1.00–1.03)	0.024
Urea (per 1 mmol/L)	1.15 (0.97–1.39)	0.104
eGFR (per 1 mL/min/1.73 m²)	0.96 (0.93–0.99)	0.012
eGFR < 60 mL/min/1.73 m²	3.68 (1.17–12.48)	0.026
LDL-C (per 1 mmol/L)	0.66 (0.40–1.04)	0.077
LVEF (per 1%)	0.99 (0.94–1.06)	0.875
CYP3A5 *1 carrier (vs *3/*3)	4.16 (1.05–15.13)	0.043
CYP3A4*22 carrier (vs CC)	1.37 (0.14–7.11)	0.747
CYP4F2 T carrier (vs AA)	1.17 (0.37–3.66)	0.781

CYP4F2 (per T allele)	0.97 (0.35–2.38)	0.951

Significant associations ($p < 0.05$) are shown in bold. CAD, coronary artery disease; CKD, chronic kidney disease; CRP, C-reactive protein; eGFR, estimated glomerular filtration rate; Hct, hematocrit; Hgb, hemoglobin; LDL-C, low-density lipoprotein cholesterol; LVEF, left ventricular ejection fraction.

S2. Baseline Differences Between CYP3A5 Genotype Groups

To assess whether the observed univariable association between CYP3A5 *1 carrier status and bleeding could be at least partly attributable to baseline imbalances between genotype groups, baseline clinical characteristics were compared across the two CYP3A5 genotype categories (Table S2). CYP3A5 *1 carriers had significantly higher prevalence of prior chronic kidney disease (30.8% versus 7.6%; p = 0.029) and of eGFR < 60 mL/min/1.73 m² (61.5% versus 29.3%; p = 0.029) than *3/*3 homozygotes. Numerically, though not statistically, they were also older and had a lower median eGFR. These imbalances motivated the multivariable adjustment described in the main manuscript (Section 3.5) and informed a cautious interpretation of the CYP3A5 association, which may be confounded by co-occurring age and renal function risk factors.

Table S2. Comparison of baseline clinical characteristics between CYP3A5 *3/*3 homozygotes and CYP3A5 *1 allele carriers. Continuous variables are presented as the median (interquartile range) and compared using the Mann–Whitney U test; categorical variables are presented as counts (percentages) and compared using the Fisher exact test.

Variable	CYP3A5 *3/*3 (n = 92)	CYP3A5 *1 carrier (n = 13)	p value
Age, years	64.0 (54.0–70.0)	66.0 (54.0–75.0)	0.596
Age ≥ 75 years	11/92 (12.0%)	4/13 (30.8%)	0.089
Male sex	69/92 (75.0%)	10/13 (76.9%)	1.000
BMI, kg/m²	25.7 (24.5–27.5)	26.3 (25.9–27.5)	0.276
eGFR, mL/min/1.73 m²	72.5 (56.8–81.7)	49.7 (42.3–70.0)	0.079
eGFR < 60	27/92 (29.3%)	8/13 (61.5%)	0.029
Creatinine, μmol/L	87.9 (79.9–102.1)	110.0 (81.5–139.6)	0.090
Hgb, g/L	144.0 (134.5–153.2)	142.0 (134.0–151.0)	0.895
CRP, mg/L	3.9 (2.2–9.6)	3.6 (1.4–6.5)	0.712
Hypertension	78/92 (84.8%)	11/13 (84.6%)	1.000
Diabetes	35/92 (38.0%)	5/13 (38.5%)	1.000
Prior CKD	7/92 (7.6%)	4/13 (30.8%)	0.029
Prior CAD	25/92 (27.2%)	5/13 (38.5%)	0.512
Current smoker	31/92 (33.7%)	6/13 (46.2%)	0.374

S3. BARC Severity-Stratified Predictor Analyses

Given the apparent severity-dependent dissociation in the CYP3A5 effect on bleeding observed in the main analysis (Section 3.6 of the main manuscript), Firth's penalized logistic regression analyses were repeated separately for minor (BARC type 1 or 2; n = 9 events) and major (BARC type 3 or 5; n = 4 events) bleeding, treated as separate outcomes. Univariable results for selected predictors are presented in Table S3 and shown in Figure S1; parsimonious multivariable models are presented in Table S4. These analyses are exploratory and should be interpreted cautiously given the very small number of events per predictor.

For minor bleeding (Table S3, left columns), CYP3A5 *1 carrier status was the strongest predictor (OR 7.54, 95% CI 1.77–32.16; p = 0.002), followed by age ≥ 75 years (OR 3.64, p = 0.026), eGFR < 60 mL/min/1.73 m^2 (OR 2.66, p = 0.038), and prior chronic kidney disease (OR 3.07, p = 0.050). For major bleeding (right columns), the pattern was markedly different: age ≥ 75 years (OR 16.71, 95% CI 2.19–127.54; p = 0.002) and prior chronic kidney disease (OR 9.74, 95% CI 1.42–66.87; p = 0.011) were the dominant predictors, whereas CYP3A5 *1 carrier status was not associated with major bleeding (OR 0.73, p = 0.214) – reflecting the complete absence of BARC 3/5 events among *1 carriers. This dissociation is consistent with the interpretation that the modest CYP3A5 association observed in the main analysis primarily reflects clinically detectable minor bleeding among CYP3A5 *1 carriers, who, in this cohort, also had a higher baseline burden of advanced age and impaired renal function.

Table S3. Univariable Firth's penalized logistic regression analyses of selected clinical and genetic predictors for minor (BARC type 1 or 2, n = 9 events) and major (BARC type 3 or 5, n = 4 events) bleeding, analyzed separately. Odds ratios (OR), 95% confidence intervals (CI), and penalized likelihood-ratio test (LRT) p values are reported. The first column pair refers to BARC minor bleeding; the second to BARC major bleeding.

Predictor	MINOR OR (95% CI)	p	MAJOR OR (95% CI)	p
Age ≥ 75 years	3.64 (0.78–14.78)	0.094	16.71 (2.51–183.17)	0.004
Prior CKD	3.07 (0.52–13.77)	0.195	9.74 (1.36–70.37)	0.025
eGFR < 60	2.66 (0.71–10.57)	0.145	4.99 (0.78–53.02)	0.089
Creatinine (per μmol/L)	1.01 (0.99–1.03)	0.287	1.02 (1.00–1.04)	0.017
CRP (per mg/L)	1.01 (0.97–1.04)	0.477	1.03 (1.00–1.06)	0.069
Hgb (per 1 g/L)	0.97 (0.93–1.01)	0.166	1.03 (0.96–1.12)	0.437
CYP3A4*22 carrier	2.11 (0.20–11.57)	0.468	1.22 (0.01–13.11)	0.898
CYP3A5 *1 carrier	7.54 (1.76–31.69)	0.008	0.73 (0.01–7.46)	0.828
CYP4F2 T carrier	1.71 (0.46–6.71)	0.423	0.55 (0.05–3.51)	0.541

Parsimonious multivariable Firth's penalized models were fitted separately for each severity (Table S4), with each model including age ≥ 75 years, eGFR < 60 mL/min/1.73 m^2, and CYP3A5 *1 carrier status. For minor bleeding, CYP3A5 *1 carrier status remained independently associated after mutual adjustment (adjusted OR 5.49, 95% CI 1.22–24.76; p = 0.008). For major bleeding, age ≥ 75 years was the dominant independent predictor (adjusted OR 13.69, 95% CI 1.76–106.42; p = 0.005), whereas CYP3A5 *1 carrier status showed only a borderline inverse association (adjusted OR 0.18, p = 0.072), reflecting the absence of major events among *1 carriers. Given the events-per-predictor ratios of approximately 3 for minor and 1.3 for

major bleeding, these severity-stratified multivariable analyses should be interpreted with caution and treated as exploratory.

Table S4. Parsimonious multivariable Firth's penalized logistic regression models were fitted separately for minor (BARC 1/2, n = 9 events; left column pair) and major (BARC 3/5, n = 4 events; right column pair) bleeding. Each model included age ≥ 75 years, eGFR < 60 mL/min/1.73 m², and CYP3A5 *1 carrier status. Given the events-per-predictor ratios of approximately 3 for minor and 1.3 for major bleeding, results should be interpreted cautiously and treated as exploratory.

Predictor	MINOR Adj OR (95% CI)	p	MAJOR Adj OR (95% CI)	p
Age ≥ 75 years	2.12 (0.36–10.99)	0.390	13.69 (1.76–164.72)	0.013
eGFR < 60	1.48 (0.29–6.92)	0.625	2.88 (0.33–34.38)	0.332
CYP3A5 *1 carrier	5.49 (1.19–24.04)	0.030	0.18 (0.00–2.60)	0.236

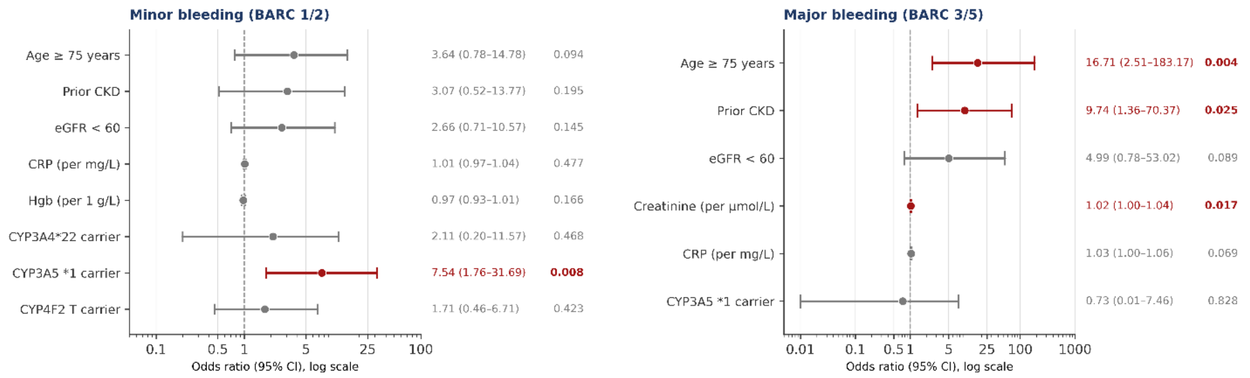

Figure S1. Side-by-side forest plots of univariable odds ratios from Firth's penalized logistic regression for minor (BARC 1/2, n = 9 events; left panel) and major (BARC 3/5, n = 4 events; right panel) bleeding. Effect estimates are shown on a logarithmic scale; the dashed vertical line indicates an OR of 1.0. Red markers and bold text denote associations significant at $p < 0.05$ (penalized likelihood-ratio test); gray markers denote non-significant associations. CYP3A5 *1 carrier status is the strongest univariable predictor of minor bleeding, whereas age and renal function are the dominant predictors of major bleeding.

Table S5. Univariable Firth's penalized logistic regression for spontaneous (non-access-site, n = 8) versus access-site (n = 5) bleeding. Odds ratios, profile penalized-likelihood 95% confidence intervals, and penalized likelihood-ratio test p values are shown. Clinical predictors are stronger for spontaneous than for access-site bleeding, indicating that the determinants of bleeding are not an artifact of femoral access.

Predictor	Spontaneous (n=8) OR (95% CI)	p	Access-site (n=5) OR (95% CI)	p
Age ≥ 75 years	7.52 (1.72–33.31)	0.008	4.63 (0.72–26.20)	0.101
eGFR < 60	3.48 (0.87–15.85)	0.078	2.95 (0.55–18.48)	0.203
Prior CKD	6.70 (1.36–30.40)	0.022	–	–
CYP3A5 *1 carrier	5.30 (1.10–23.25)	0.039	2.36 (0.22–14.22)	0.416

S4. Machine-Learning Analysis and Feature Importance

To complement the regression analysis with a non-linear perspective and quantify the incremental discriminative value of genotype information beyond clinical variables alone, several XGBoost gradient-boosted tree classifiers were trained using stratified 5-fold cross-validation, repeated 20 times, yielding 100 out-of-fold area under the ROC curve (AUC) estimates per model configuration.

The parsimonious clinical model (age, eGFR, and prior chronic kidney disease) achieved a mean AUC of 0.633 (SD 0.167; median 0.657). Adding CYP3A5 *1 carrier status yielded a mean AUC of 0.634 (SD 0.175), which was not significantly different from that of the clinical model (paired Wilcoxon $p = 0.95$). A broader clinical model including eight features (age, sex, diabetes, hypertension, hemoglobin, prior coronary artery disease, prior chronic kidney disease, and eGFR) achieved the highest mean AUC of 0.704 (SD 0.167). Further addition of CYP3A5 *1 carrier status did not improve discrimination (mean AUC 0.701; $p = 0.35$). Out-of-fold ROC curves are shown in Figure S2.

SHAP (SHapley Additive exPlanations) analysis of the clinical + CYP3A5 XGBoost model (Figure S3) identified eGFR and age as the two most influential features driving predicted bleeding risk, with CYP3A5 *1 carrier status ranking third. The cross-validated AUC distributions across all model configurations are shown in Figure S4. The absence of measurable improvement in XGBoost AUC after adding CYP3A5 – despite its univariable significance – is consistent with the partial correlation between CYP3A5 *1 carrier status and other renal and age-related predictors (Table S2) and supports the interpretation that the CYP3A5 association in the main analysis is, at least in part, confounded by these co-occurring clinical risk factors.

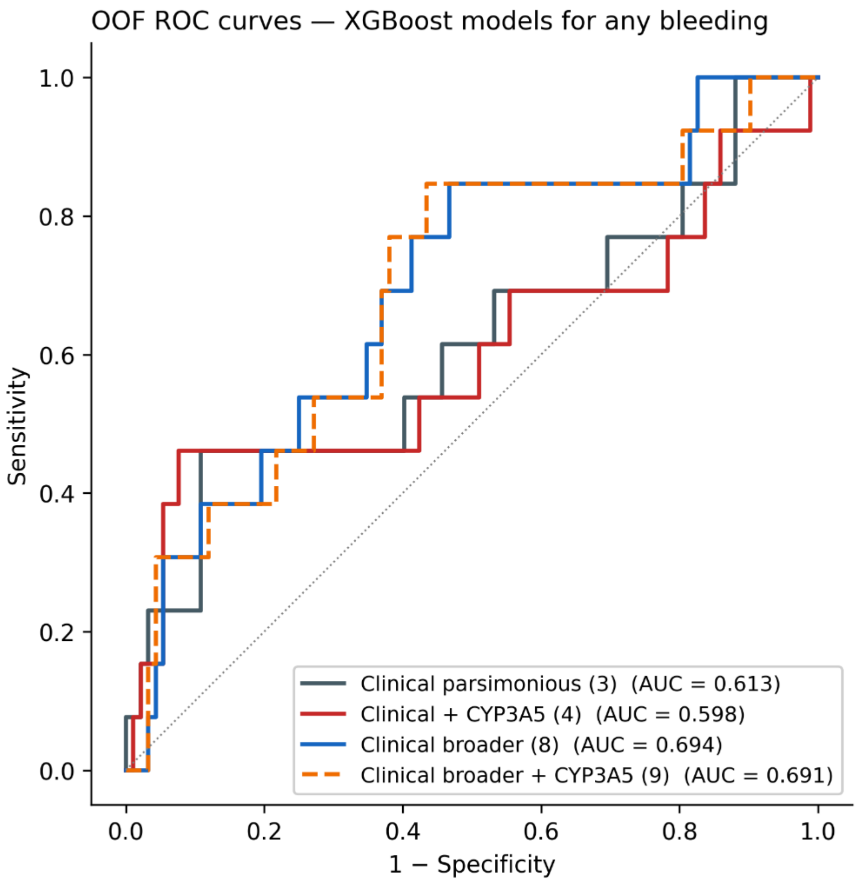

Figure S2. Out-of-fold receiver-operating-characteristic (ROC) curves for four XGBoost model configurations predicting any bleeding, constructed from pooled out-of-fold predictions across 5-fold cross-validation repeated 20 times. The diagonal grey dotted line represents the theoretical chance line.

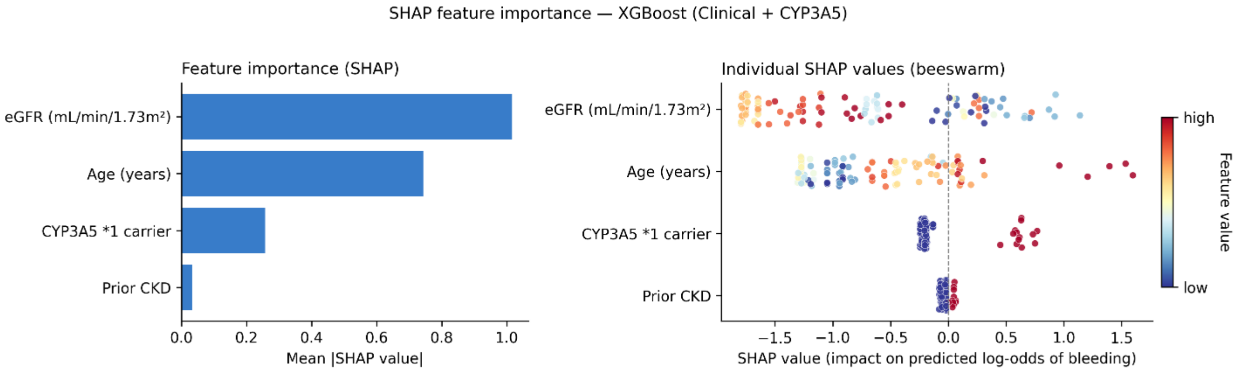

Figure S3. SHAP (SHapley Additive exPlanations) analysis of the XGBoost model combining the parsimonious clinical predictor set with CYP3A5 *1 carrier status. Left panel: mean absolute SHAP values, quantifying each feature's overall importance. Right panel: individual SHAP values (beeswarm plot) for all 105 patients, showing the direction and magnitude of each feature's contribution to the predicted log-odds of bleeding. Color encodes the relative magnitude of the feature value (red = high, blue = low). The vertical dashed line denotes a SHAP value of zero.

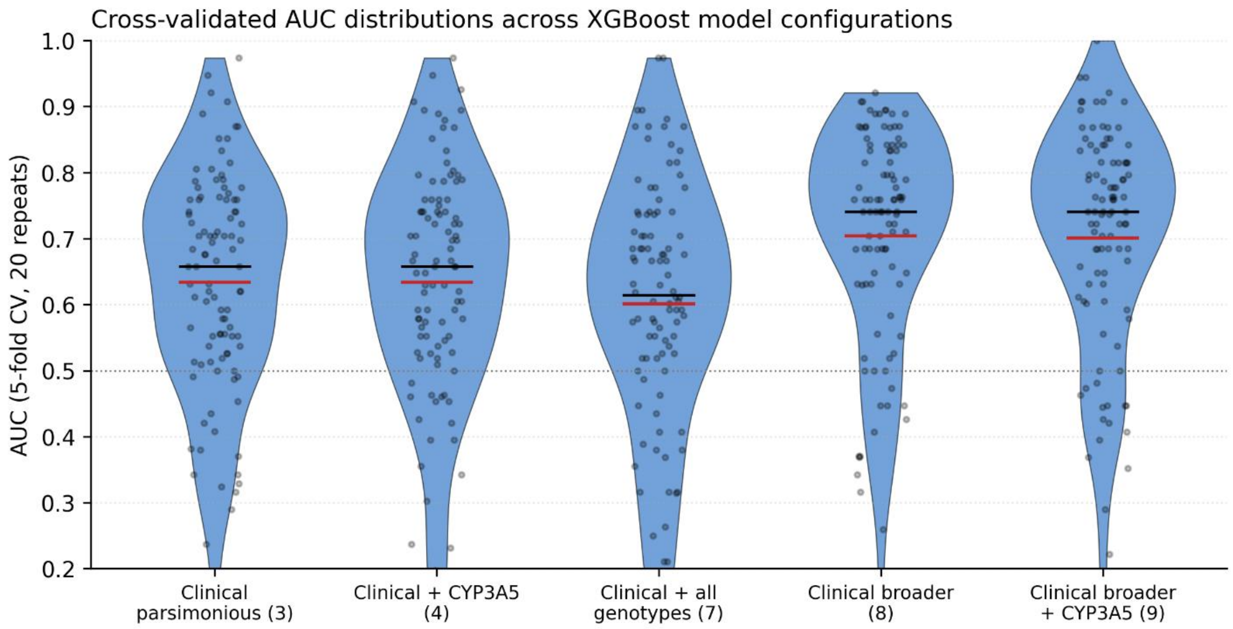

Figure S4. Cross-validated out-of-fold AUC distributions across five XGBoost model configurations (5-fold cross-validation repeated 20 times, yielding 100 AUC estimates per configuration). Violin plots show the full distribution of AUC values for each model; red horizontal lines indicate the mean AUC; black horizontal lines indicate the median; individual points represent each of the 100 cross-validation folds. The gray dotted horizontal line represents the theoretical chance level (AUC = 0.5).

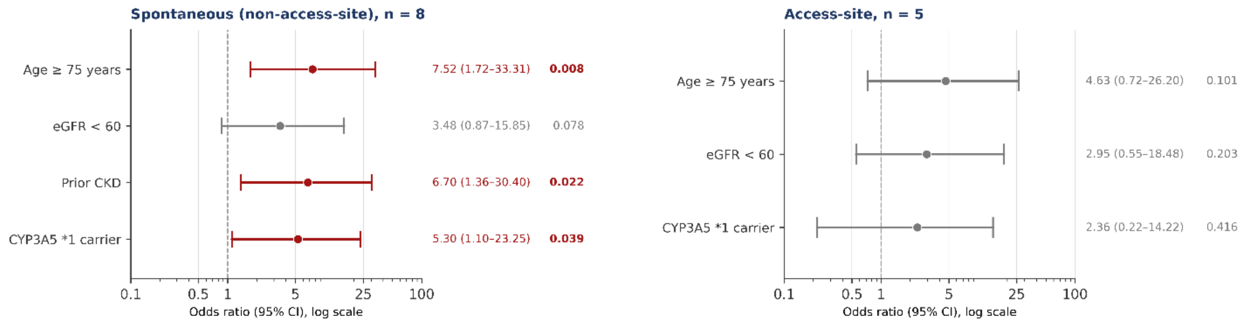

Figure S5. Forest plots of univariable odds ratios for spontaneous (non-access-site, n = 8; left) versus access-site (n = 5; right) bleeding, estimated by Firth's penalized logistic regression. Estimates are plotted on a logarithmic scale; the dashed line denotes an odds ratio of 1.0. Points are odds ratios and horizontal bars the profile penalized-likelihood 95% confidence intervals; p values are from the penalized likelihood-ratio test. Significant associations ($p < 0.05$) are shown in red, non-significant ones in grey. CKD, chronic kidney disease; eGFR, estimated glomerular filtration rate.
